# Pomegranate Peel as a Sustainable Additive for Baijiu Fermentation: Physicochemical and Flavor Analysis with Process Optimization

**DOI:** 10.3390/molecules30081800

**Published:** 2025-04-17

**Authors:** Longwen Wang, Guida Zhu, Na Li, Zhiheng Wang, Yi Ji, Chen Shen, Jing Yu, Ping Song

**Affiliations:** 1 School of Food Science and Pharmaceutical Engineering, Nanjing Normal University, Nanjing 210023, China; 232712052@njnu.edu.cn (L.W.); 232702011@njnu.edu.cn (G.Z.); 232712046@njnu.edu.cn (Z.W.); 28230130@njnu.edu.cn (Y.J.); 2 Huaguan Group Brewery Co., Ltd., Heze 274900, China; 15020470277@163.com; 3 Shimadzu (China) Co., Ltd., Shanghai 200233, China; sshshenc@shimadzu.com.cn

**Keywords:** fresh pomegranate peel, rice hull, fermentation, light-flavor Baijiu, quality Baijiu

## Abstract

Rice hulls, a traditional ingredient in Chinese light-flavor Baijiu, contribute to bran and furfural flavors but may adversely affect the aroma and taste. This study explores fresh pomegranate peel as a sustainable alternative to rice hulls in Baijiu fermentation. The flavor profiles in *jiupei* and Baijiu were interpreted by employing head-space solid-phase microextraction coupled with gas chromatography–mass spectrometry (HS-SPME-GC-MS), while their physicochemical characteristics were systematically assessed. Statistical evaluations, such as correlation analysis and cluster analysis, were conducted to interpret the data. The results showed that compared with rice hull, pomegranate peel reduced furfural content in *jiupei* by 90%, increased the alcohol distillation rate (alcohol distillation rate: this refers to the weight percentage of 50% alcohol by volume (ABV) Baijiu produced from a unit amount of raw material under standard atmospheric pressure at 20 °C (also known as Baijiu yield)) by 30%, enhanced antioxidant capacity by 24.38%, and improved starch efficiency by 3%. Notably, the Baijiu complied with the premium Baijiu standards specified in the Chinese National Standard for light-flavor Baijiu. Additionally, under the experimental conditions of this study, the optimal Baijiu yield (optimal Baijiu yield: the maximum achievable Baijiu production under defined constraints (e.g., energy input, time, cost)) (48% ± 3.41%) correlated with the pomegranate peel particle size. This research highlights the viability of using pomegranate peel as a sustainable and environmentally friendly adjunct in the fermentation of light-flavor Baijiu, offering valuable perspectives for exploring alternative brewing ingredients.

## 1. Introduction

Among the four classic varieties of traditional Chinese Baijiu, light-flavor Baijiu holds a significant place [1]. Light-flavor Baijiu is distinguished by its clean and refined flavor profile, featuring a smooth, sweet, and delicate taste; balanced natural harmony; and a refreshing finish, earning recognition for its elegant and pure sensory qualities [2]. Rice hull serves as a vital adjunct in the production of light-flavor Baijiu, providing structural support, enhancing porosity, and ensuring proper aeration during the brewing process. It also facilitates the cooking and distillation of *jiupei*, as well as promotes saccharification and fermentation. However, the distinctive brewing method of light-flavor Baijiu, which involves a high proportion of rice hulls (15~20%), often results in the introduction of unwanted aromas, including chaff, furfural, and moldy notes, into the Baijiu [3]. Furthermore, the elevated levels of gliadin and polysaccharides in rice hull tend to generate methanol and furfural during fermentation, which are primary contributors to the chaff-like flavor often found in light-flavor Baijiu. Thus, investigating alternative brewing adjuncts for Baijiu production and examining their impact on the fermentation process is crucial to achieving a refined balance of flavor and texture.

The outer layer of the pomegranate, belonging to the Punicaceae family, is abundant in numerous bioactive compounds, including polyphenols, flavonoids, anthocyanins, and organic acids. These compounds contribute to its diverse biological activities, such as antioxidant, anti-inflammatory, and cardioprotective features [4,5]. Pomegranate peel has also been applied in functional yogurt production, where its extracts enhanced the total phenols, flavonoids, anthocyanins, and antioxidant activity of yogurt, which is of great nutritional value [6]. In addition, pomegranate peel has also been used in polymer scaffolds for bone regeneration, greatly improving the porosity and support strength of the polymer scaffolds [7]. However, globally, approximately 1.9 million metric tons of pomegranate peel remain unprocessed and underutilized, except for limited medicinal applications [8], resulting in significant resource wastage, with few reports documenting its utilization in Baijiu production.

This study examined the use of pomegranate peels with varying granular dimensions (compared to standard rice hull) as adjunct materials to evaluate their impact on light-flavor Baijiu production. The physical and chemical qualities and flavor profiles of *jiupei* during fermentation were assessed using gas chromatography–mass spectrometry (GC-MS), conventional analytical approaches, and multivariate statistical analysis. By integrating the yield and physical and chemical properties of Baijiu, a comprehensive investigation into the use of adjuncts in light-flavor Baijiu production was conducted. The findings not only validate the suitability of pomegranate peel as a brewing adjunct for light flavor Baijiu but also offer worthwhile insights for exploring alternative adjunct materials in its production.

## 2. Results and Discussion

### 2.1. Physical and Chemical Properties of Jiupei

#### 2.1.1. Dry Mass

**Figure 1:**
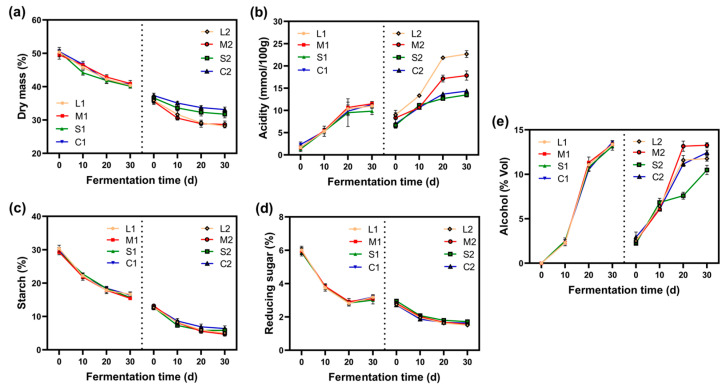
Changes in physicochemical properties of (**a**) dry mass, (**b**) acidity, (**c**) starch, (**d**) reducing sugar, and (**e**) alcohol. The left side represents the first fermentation stage, and the right side is the second fermentation stage. C1 and C2 represent the control groups (rice hulls), whereas L1, M1, S1, L2, M2, and S2 represent the experimental groups (the particulate size of fresh pomegranate peel ranging from 0.5 to 2.5 cm). These are defined in Table 1.

Figure 1a illustrates the dry mass variation of *jiupei* during two fermentation stages. A progressive decreasing trend in dry mass can be observed. During the first fermentation stage, the dry mass decreased from approximately 50% to around 40%. At the onset of the secondary fermentation stage, the dry mass was lower than the terminal value of the primary stage, likely attributed to additional moisture introduced during *jiupei* distillation, which could relatively decrease the dry mass based on the measurement methodology. Alternatively, certain soluble substances (e.g., minerals and salts) might dissolve into the liquid phase during distillation, becoming slightly entrained by steam or retained in equipment, thereby contributing to dry mass loss. In the secondary fermentation stage, the dry matter further decreased by approximately 6%, reaching 27–33%. Overall, the principal cause of dry mass reduction lies in diverse microbial activities and associated biochemical reactions during fermentation [9]. Notably, at the conclusion of secondary fermentation, samples containing pomegranate peels exhibited 2–5% lower dry mass compared with rice hulls containing samples, resulting from the combined effects of pomegranate peels’ 50% lower initial dry mass than rice hulls and related microbial metabolic activities.

#### 2.1.2. Acidity

As illustrated in Figure 1b, the acidity of *jiupei* exhibited an overall increasing trend during both fermentation stages. Compared to the first fermentation stage, the differences among experimental groups in the second fermentation stage were more pronounced, particularly in samples containing medium- (1.5 × 0.3 × 0.3 cm^3^) and larger (2.5 × 0.3 × 0.3 cm^3^)-sized pomegranate peels, which showed the most significant changes in acidity. This manifestation may be attributed to the fact that pomegranate peels promote the substantial growth of certain anaerobic or facultative anaerobic microorganisms, such as lactic acid bacteria, thereby enhancing acidity [10]. Furthermore, variations in acidity were also observed among samples with different sizes of pomegranate peels, highlighting the importance of the granular dimension of auxiliary materials.

#### 2.1.3. Starch and Reducing Sugar Content

Fermentation begins with the microbial breakdown of starch into reducing sugars, which are subsequently converted into alcohols, acids, and flavor compounds [11]. The starch concentration serves as an indicator of fermentation efficiency and Baijiu yield. As shown in Figure 1c, the starch level, in all samples reduced after both the first and second fermentation stages of *jiupei*, exhibiting a stepwise decline. In Figure 1d, a slight increase in the reducing sugar content was observed during the later phase of the first fermentation stage, which may be attributed to the hydrolysis of residual starch during that period, leading to a marginal rise in reducing sugar levels [12].

On top of this, the choice of auxiliaries also influenced the starch content in *jiupei*. During the second fermentation stage, the M2 and L2 samples exhibited the highest starch utilization rates, suggesting that pomegranate peels, particularly when ground into larger particles, enhance the ability of lactic acid bacteria to metabolize starch (starch utilization experienced a 3% rise). By way of example, α-amylase in fermented grains cleaves the α-1,4 glycosidic bonds in starch, generating dextrins of varying molecular weights. Simultaneously, it rapidly liquefies small amounts of maltose and glucose, ultimately converting starch into maltose, glucose, and isomaltose.

When glucoamylase degrades starch, the resulting reducing sugars are consumed by yeast, promoting yeast growth, reproduction, and fermentation activity [13]. This is consistent with our findings.

#### 2.1.4. Alcohol Content

During the fermentation process, Saccharomyces cerevisiae rapidly proliferates, producing a significant amount of alcohol as a metabolic byproduct [14]. Consequently, fluctuations in alcohol content throughout the fermentation process reflect the growth and metabolic activities of Saccharomyces cerevisiae within *jiupei*. As illustrated in Figure 1e, after 30 days of fermentation, the alcohol content increased by 13–14% during the first fermentation stage and by 8–12% during the second fermentation stage. This discrepancy arises because the first fermentation stage represents a period of intense anaerobic alcoholic fermentation by yeast.

In contrast, during the second fermentation stage, the high acidity and elevated alcohol concentration in the environment inhibited the growth, reproduction, and metabolic activities of yeast. Notably, when fresh pomegranate peels had medium-sized particles (1.5 × 0.3 × 0.3 cm^3^), the distilled alcohol content in the *jiupei* was the lowest (at the onset of the second fermentation), indicating that a considerable proportion of alcohol compounds had volatilized.

#### 2.1.5. Flavor Content

**Figure 2:**
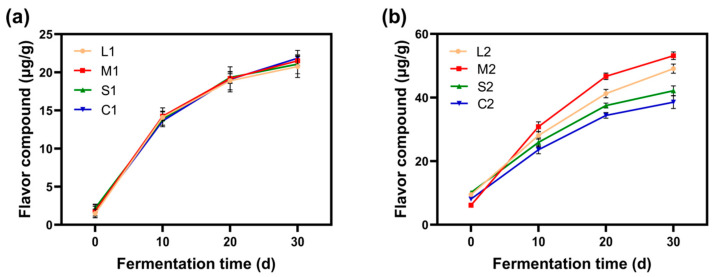
Changes in the content of flavor substances (**a**) of *jiupei* in the first fermentation and changes in the content of flavor substances (**b**) of *jiupei* in the second fermentation.

Figure 2a,b depict the changes in flavor content during the fermentation process of *jiupei*. Prior to the first fermentation stage, the concentration of volatile flavor compounds entering the fermentation vessel was only 1–3 μg/g; however, after 30 days, the concentration surged to 20–23 μg/g. During the second fermentation stage, the content of flavor compounds was significantly higher than that in the first fermentation stage due to the addition of fresh pomegranate peels [15].

Furthermore, the optimal grinding size enhances the porosity of *jiupei*, facilitating the extraction and generation of flavor compounds (as demonstrated by M2). As the particle size of pomegranate peel decreases, the reactive surface area increases but causes the porosity of *jiupei* to first increase and then decrease, thereby affecting the generation and volatilization of flavor compounds during the second fermentation stage. The flavor compounds in *jiupei*, which are metabolic products of microorganisms, play a vital role in determining the flavor profile of Baijiu [16]. Notably, the use of fresh pomegranate peels during the steaming of *jiupei* significantly increases the content of key flavor compounds in Baijiu, including ethyl acetate, ethyl butyrate, α-ketoglutaric acid, acetic acid, isoamyl alcohol, 3-hydroxy-2-butanone, 2-methyl-1-butanol, and geranylacetone (see Tables S1–S3; additional data are provided in the Supplementary Materials).

#### 2.1.6. Association Analysis

The Pearson correlation coefficient is a statistical measure used to quantify the degree of linear correlation between two continuous variables, with values ranging from −1 to 1 [17,18]. It plays a critical role in analyzing datasets that approximate a normal distribution. To investigate the relationships between the physicochemical properties of *jiupei*, this study employed the Pearson correlation coefficient to evaluate their interdependencies.

**Figure 3:**
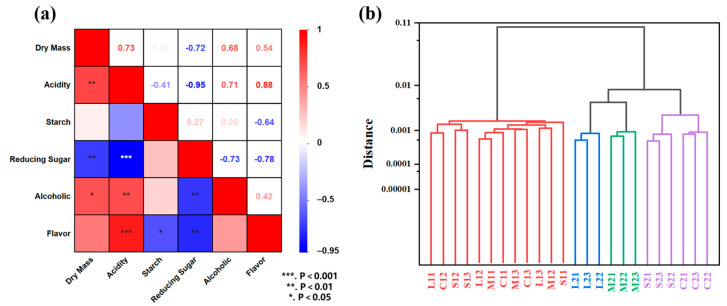
(**a**) Association analysis of the physicochemical properties; (**b**) cluster analysis of physical and chemical properties.

As illustrated in Figure 3a, a substantial negative correlation (*p* < 0.01) exists between the dry mass and reducing sugar concentration in *jiupei*, suggesting that elevated dry mass may accelerate microbial consumption of reducing sugars through enhanced hydrolysis and metabolic processes. Conversely, the positive correlations of dry mass with acidity (*p* < 0.01) and alcohol concentration (*p* < 0.01) indicate that a greater dry mass facilitates the enzymatic hydrolysis of starch (generating organic acids) and yeast-mediated alcoholic fermentation. The negative correlations between reducing sugar content and dry mass, alcohol concentration, acidity, and flavor compounds (*p* < 0.01) reflect substrate depletion dynamics, wherein rapid sugar conversion simultaneously drives acid accumulation, ethanol synthesis, and flavor precursor formation. In addition, a highly positive correlation between acidity and flavor compounds (*p* < 0.001) reveals acid-catalyzed esterification as a critical pathway for flavor compounds during solid-state fermentation.

### 2.2. Effect of Pomegranate Peel on the Physical and Chemical Properties of Jiupei

#### Cluster Analysis

Substances exhibiting correlations can be categorized into distinct groups, and therefore, we conducted a cluster analysis on the physical and chemical properties of *jiupei*. The results of the analysis, performed using the between-group linkage method, are presented in Figure 3b. Based on the Euclidean distance of 0.002, the data can be divided into four distinct clusters. *Jiupei* from the first fermentation stage belongs to the first cluster, while L2 from the second fermentation stage falls into the second cluster, M2 falls into the third cluster, and S2 and C2 fall into the fourth cluster. This indicates that auxiliary materials with grinding granularities of S and C exert nearly identical effects to *jiupei*, such as in terms of porosity and resistance to compression.

### 2.3. Physical and Chemical Properties of Light-Flavor Base Baijiu

#### 2.3.1. Solids Content

**Figure 4:**
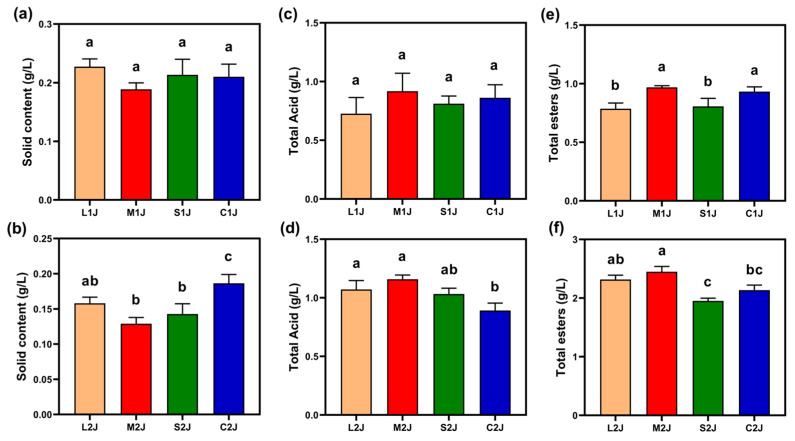
Contents of (**a**,**b**) solids, (**c**,**d**) total acids, and (**e**,**f**) total esters. The top images represent the first fermentation stage, and the bottom images indicate the second fermentation stage. C1J and C2J represent the control groups (rice hull), whereas L1J, M1J, S1J, L2J, M2J, and S2J represent the experimental groups (the particulate size of fresh pomegranate peel ranging from 0.5 to 2.5 cm). These are defined in Table 1. The letters a, b, and c in the center of the figure represent symbols of differences. Different letters indicate significant differences between two groups, while the same letters indicate no significant differences between two groups. Letter combination (e.g., ab) indicates no significant differences between ab and either a or b.

Baijiu solids are nonvolatile organic substances in Baijiu, which are produced through fermentation and include alcohols, esters, acids, aldehydes, and ketones [19]. Excessive Baijiu solids (>0.4 g/L) cause Baijiu to lose light, turbidity, and precipitation, affecting product quality [20]. As shown in Figure 4a,b, it can be seen that the solids content of the first fermented Baijiu was below 0.3 g/L, fully meeting the requirements of the Chinese national standard for solids content in light-flavor Baijiu [21] (≤0.5 g/L), while the M1J group had the lowest solids content, probably because pomegranate peels of this size (1.5 × 0.3 × 0.3 cm^3^) reduce the entry of difficult-to-volatilise, high-boiling-point, high-level esters, acids, and alcohols into the Baijiu when distilling Baijiu. The solids content of the second fermented Baijiu was below 0.20 g/L, which was lower than that of the first fermented Baijiu, probably because the first fermentation consumed more initial substances such as starch and reducing sugar in the sorghum, which led to a decrease in the solids of the nonvolatile, high-boiling-point, high-level esters, acids, and alcohols formed by the fermentation.

#### 2.3.2. Total Acid Content

Organic acids in Baijiu play a vital role in shaping its flavor profile, imparting a rich, smooth, and well-rounded taste [22]. Additionally, these acids help mitigate bitterness, enhancing the overall sensory experience and elevating the quality of the Baijiu. As illustrated in Figure 4c,d, the M1J group from the first fermentation exhibited the highest total acid content, reaching 0.92 g/L. This meets the Chinese national standard for premium light-flavor Baijiu [21], which requires a total acid content of at least 0.5 g/L. This outcome may be attributed to the optimal supporting effect of medium-sized pomegranate peel particles (1.5 × 0.3 × 0.3 cm^3^) in *jiupei*, thereby facilitating the evaporation of volatile organic acids (such as acetic acid, propionic acid, butyric acid, and decanoic acid) from *jiupei* during distillation.

In the second fermentation, the total acid content of Baijiu increased by 0.20–0.46 g/L compared to the first fermentation. This rise is likely, which suggests the increased porosity provided by pomegranate peel or rice hulls, which created more space between sorghum grains. This enhanced environment allowed microorganisms to thrive and perform metabolic activities more efficiently, resulting in higher acid production.

#### 2.3.3. Total Ester Content

Esters are key flavor compounds in Baijiu, typically imparting fruity notes that enhance its aroma and contribute to a pleasant, well-balanced taste [23]. They also play a significant role in modulating the overall flavor profile of Baijiu. As shown in Figure 4e,f, the M1J and C1J groups from the first fermentation exhibited the highest total ester content, both exceeding 0.93 g/L. This meets the Chinese national standard for superior light-flavor Baijiu [21], which requires a minimum total ester content of 0.80 g/L. These results suggest that pomegranate peel of the specified size (1.5 × 0.3 × 0.3 cm^3^) can achieve ester levels comparable to those of rice hull (size of 0.8 × 0.33 × 0.28 cm^3^), indicating its effectiveness in evaporating esters during the steaming process when used as a substitute for rice hull.

In the second fermentation, the total ester content across all four groups ranged from 1.95 to 2.45 g/L, substantially higher than in the first fermentation. This increase may be attributed to the combined action of pomegranate peel or rice hull with sorghum, which facilitates microbial activity by other organisms, such as yeasts, leading to enhanced ester production.

#### 2.3.4. Total Phenol Content

Total polyphenols are compounds characterized by multiple phenolic hydroxyl groups attached to a benzene ring structure [24,25]. These molecules can donate hydrogen protons and form stable semiquinone radicals when reacting with peroxyl radicals, thereby interrupting free radical chain reactions. This mechanism enables them to effectively scavenge free radicals and inhibit lipid peroxidation, resulting in strong antioxidant properties.

**Figure 5:**
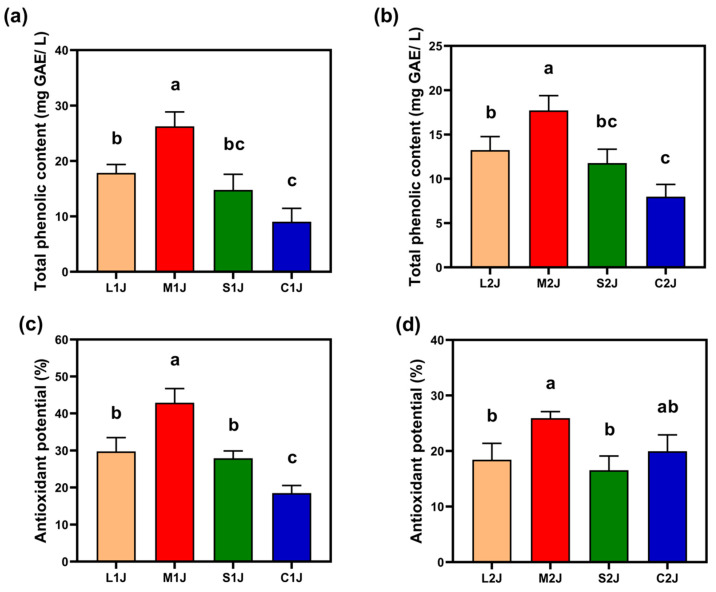
Contents of (**a**,**b**) total phenolic content. (**c**,**d**) Antioxidant capacity analyses. The left image represents the first fermentation stage, and the right image represents the second fermentation stage. C1J and C2J represent the control groups (rice hull), whereas L1J, M1J, S1J, L2J, M2J, and S2J represent the experimental groups (with the particulate size of fresh pomegranate peel ranging from 0.5 to 2.5 cm). These are defined in Table 1. Letters a, b, and c in the center of the figure represent symbols of differences. Different letters indicate significant differences between two groups, while the same letters indicate no significant differences between two groups. Letter combination (e.g., ab) indicates no significant differences between ab and either a or b.

As depicted in Figure 5a,b, the total phenol content in the M1J, L1J, and S1J groups of the first fermented Baijiu was higher than that of the C1J group. This difference can be elucidated by the evaporation of phenolic compounds from pomegranate peels during the steaming process, which increased the total phenol content compared to the C1J group. In addition, variations in the size of the pomegranate peels contributed to differences in phenol levels. This phenomenon may be related to the porosity of *jiupei* and the degree of pomegranate peel cell disruption. Both large and small pomegranate peel particles may exhibit lower porosity in *jiupei* compared to medium-sized particles. Additionally, insufficient cell disruption in large particles may hinder complete phenolic release, whereas excessive disruption in small particles could trigger premature degradation of phenolics by polyphenol oxidase (PPO).

Similarly, in the second fermentation, the total phenol content in the M2J, L2J, and S2J groups was also higher than that of the C2J group. However, the phenol content in the second fermentation was lower than in the first fermentation, likely due to the reduced quantity of pomegranate peel used as an adjunct.

#### 2.3.5. Antioxidant Capacity

Pomegranate peel possesses bioactive properties that confer health benefits, with antioxidant activity being a key feature [26]. This capacity is crucial for neutralizing free radicals, reducing reactive oxygen species, and mitigating the risk of cardiovascular diseases. As illustrated in Figure 5c,d, the M1J group from the first fermentation exhibited the highest DPPH-scavenging ability, indicating superior antioxidant activity compared to other groups. This is likely due to the M1J group’s elevated total phenol content, which correlates with enhanced antioxidant performance.

In contrast, the antioxidant capacity of the second fermented Baijiu was lower than that of the first fermentation. This decline may be attributed to the reduced quantity of pomegranate peel used as an adjunct, which aligns with the observed decrease in total phenol levels.

#### 2.3.6. Yield of Baijiu

**Figure 6:**
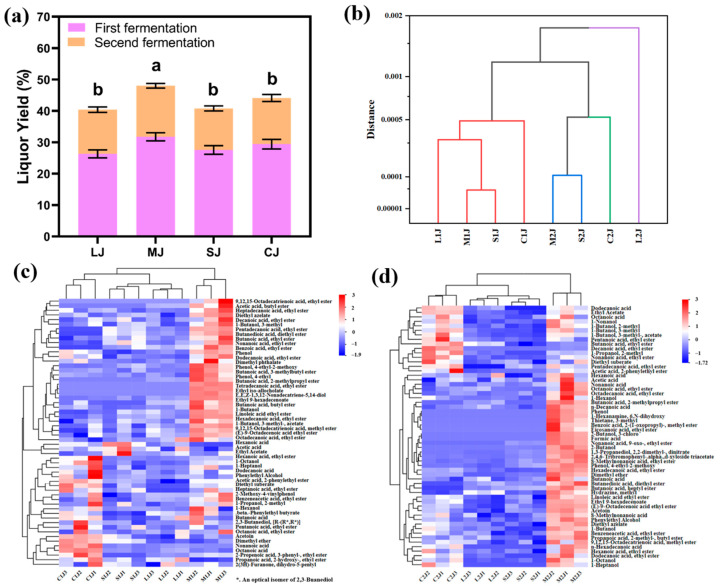
Comparative analysis of Baijiu yield under different fermentation schemes (**a**). Cluster analysis of physicochemical properties of light-flavor base Baijiu (**b**). Cluster heatmap of volatile compounds in double-fermentation Baijiu (**c**,**d**). The content of metabolites is represented according to the color scale in the upper right corner, as reflected in each rectangle of the heatmap. Darker red corresponds to higher compound content, while darker blue corresponds to lower compound content. Different letters indicate significant differences between two groups, while the same letters indicate no significant differences between two groups.

As illustrated in Figure 6a, the yield of pomegranate peel Baijiu after the first fermentation stage ranged between 26% and 32%, with some samples exceeding the yield of the control group using 15% rice hull. As the particle size of the pomegranate peels increased, the yield initially rose and then declined, with the M group achieving the highest yield of 32%.

During the second fermentation stage, the Baijiu yield varied between 13% and 17%. Granular pomegranate peel in the M particle size group exhibited the highest Baijiu yield, followed by the L particle size group and the control group with 15% rice hull (C).

#### 2.3.7. Effect of Pomegranate Peel on the Physical and Chemical Properties of Base Baijiu

As demonstrated in the group analysis of base Baijiu in Figure 6b, samples with a Euclidean distance of 0.0005 were divided into four types using the between-group linkage method. During the first fermentation stage, all Baijiu samples belonged to the first type. In the second fermentation stage, Baijiu from the M2J and S2J groups was classified into the second category, C2J was classified into the third category, and L2J was classified into the fourth type. These clustering results are largely consistent with the physical and chemical qualities of Baijiu shown in Figure 3b, illustrating that the quality of base Baijiu can be accurately assessed based on the physical and chemical properties of *jiupei* and fermentation conditions.

As an essential component of Chinese baijiu [27], light-flavor Baijiu is highly favored by consumers due to its distinctive floral and fruity aromas [28]. Therefore, research on light-flavor Baijiu holds significant importance. As shown in Table S3 (additional data provided in the Supplementary Materials), replacing rice hull with fresh pomegranate peels as an auxiliary material significantly reduces the furfural content in base Baijiu, with higher furfural content tending to impart undesirable off-flavors, such as rice hull-like flavors, to the Baijiu.

#### 2.3.8. Flavor Analysis of Light-Flavor Base Baijiu

Figure S1a,b (additional data are provided in the Supplementary Materials) highlight the variations in the number of flavor compounds across different base Baijiu samples. During the first fermentation, 76 to 98 flavor compounds were identified, predominantly consisting of alcohols, esters, and acids. Among these, Baijiu M and L contained the fewest types of aldehydes. In the second fermentation, the number of flavor compounds increased to 91–117. While the quantities of alcohols and acids remained relatively stable between the two fermentations, the number of esters rose significantly during the second fermentation. Furthermore, substituting fresh pomegranate peel for rice hull after the first fermentation and before distillation increases the quantity of flavor compounds.

Figure S1c,d (additional data are provided in the Supplementary Materials) illustrate the variations in the concentrations of flavor compounds among various base Baijiu samples. Baijiu M1J exhibited the highest content of flavor compounds, followed by S1J and C1J, while L1J showed minimal content. Alcohols and esters were the predominant flavor compounds in Baijiu after the first fermentation stage, with the concentrations of other compounds being relatively low. The porosity of fermented and distilled Baijiu influenced the categories and concentrations of flavor compounds, which varied depending on the auxiliary materials used.

#### 2.3.9. Impact of Pomegranate Peels on the Flavor of Light-Flavor Base Baijiu

As depicted in Figure 6c,d and Tables S1–S8 (additional data are provided in the Supplementary Materials), the major esters identified in pomegranate peel-based Baijiu during both fermentation stages included ethyl acetate, ethyl butyrate, butyl butyrate, n-butyl acetate, ethyl hexanoate, ethyl nonanoate, diethyl azelate, ethyl laurate, ethyl palmitate, ethyl myristate, ethyl 9-hexadecenoate, ethyl elaidate, and ethyl linoleate (the red regions in the heatmap). These esters are primarily formed through the esterification of acids and alcohols, the non-oxidative ethanol metabolic pathway in Saccharomyces cerevisiae and other fungi, and the esterase pathway [29,30].

Except for ethanol, the main alcohols detected in pomegranate peel-based Baijiu were isoamyl alcohol, 2-methyl-1-butanol, phenylethyl alcohol, n-octanol, n-nonanol, linalool, and 2-nitroethanol (the red regions in the heatmap). These alcohols are mainly produced through the reduction of amino acids by yeast under anaerobic conditions [31], the conversion of sugars under aerobic conditions, or the reduction of corresponding aldehydes [32,33]. Notably, linalool is considered to substantially contribute to the floral aroma of light-flavor Baijiu.

Following the addition of pomegranate peels, the key aldehydes and ketones in the base Baijiu included 3-hydroxy-2-butanone, geranylacetone, (Z)-oxocyclopent-6-en-2-one, and coconut aldehyde, with their concentrations varying significantly among different base Baijiu samples. Geranylacetone, known for its rosy and fruity notes, is also an important flavor component in tea [34].

The addition of fresh pomegranate peel showed a lower furfural content in the base Baijiu compared with rice hull, likely due to changes in the fermentation environment, nutritional composition, and microbial metabolic activity induced by the pomegranate peels. Higher levels of 4-ethylguaiacol, 4-ethyl-2-methoxyphenol, dimethyl ether, fluoropropylene oxide, and 1,3-bis(cinnamoyloxymethyl)adamantane were observed in the M1J and M2J base Baijiu samples. Previous studies have shown that 4-methylguaiacol and 4-ethylguaiacol in Baijiu exhibit in vitro antioxidant activity [35]. This is consistent with the enhanced antioxidant capacity observed in pomegranate peel-based Baijiu. Furthermore, the concentration of 1-ethoxy-1-methoxyethane in the base Baijiu was raised with the addition of pomegranate peels.

## 3. Materials and Methods

### 3.1. Chemicals and Reference Materials

All chemicals, including phenolphthalein, sodium hydroxide, copper sulfate pentahydrate, methylene blue, sodium potassium tartrate, potassium ferrocyanide, zinc acetate, acetic acid, glucose, sodium chloride, sodium carbonate, gallic acid, and DPPH (analytical grade), were sourced from Alfa Aesar in Heysham, Lancashire, United Kingdom. Chromatography-grade 2-octanol was obtained from TCI Chemicals in Chuo-ku, Tokyo, Japan. Sorghum was procured from Yixing Yihong Rice Industry in Wuxi, China, while fresh Mengzi pomegranate was supplied from Yunnan June Red Agricultural Technology Co., Ltd., Mengzi, China. Fresh pomegranates were transported to the laboratory under low-temperature conditions. Upon arrival, the fresh fruits were hermetically sealed in fresh-keeping bags and cryopreserved at −20 °C for subsequent experiments. The pomegranate peels used in this investigation originated from Mengzi pomegranates, with controlled exfoliation approaching distillation stage to retain the three-layer pericarp structure (exocarp, mesocarp, and internal chamber septa). The separated peels were preserved in sealed bags under 4 °C refrigeration.

### 3.2. Apparatus and Tools

The equipment utilized in this study included an oven (DHG-9240A; Shanghai Yiheng Technology Instrument, Shanghai, China), a rotary evaporator (B-100 HB; BUCHI Operations India Private Limited, Sachin INA, India), an electric furnace (FD-1000W; Changsha Miqi Instrument and Equipment, Changsha, China), a microplate reader (JC-1200MB; Qingdao Jingcheng Instrument and Meter, Qingdao, China), an autoclave (GI80T; ZEALWAY INSTRUMENT Inc., Wilmington, DE, USA), an electronic balance (BSA124S; Sartorius, Göttingen, Germany), and a gas chromatography–mass spectrometry system (5977C GC/MSD Agilent Technologies, Santa Clara, CA, USA).

### 3.3. Methodology

#### 3.3.1. Production of Light-Flavor Baijiu

**Figure 7:**
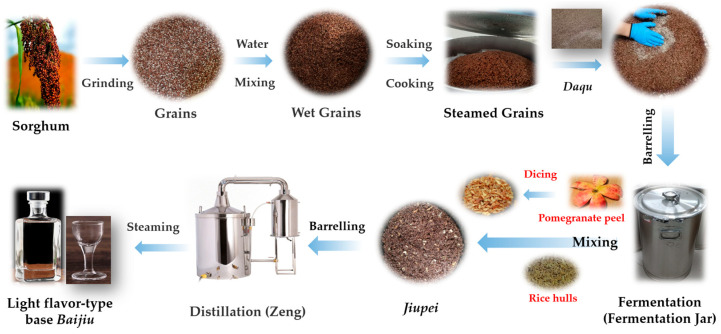
Brewing process of light-flavor Baijiu.

As depicted in Figure 7 [36,37,38], barrel fermentation begins with grinding sorghum to ensure uniformity of the grains after fermentation. The sorghum for each experimental group is processed identically, involving crushing, drying, and mixing. Following this, 6 kg of ground sorghum (excluding the barrel weight) is placed into a 35 L stainless steel fermentation barrel. The study comprises four experimental groups, each with three replicates. During Phase Two, the crushed sorghum is hydrated with water (60% of the weight relative to the raw material) at 80 °C and left to rest for 20 h. The third stage consists of steaming the sorghum for 60 min. In the fourth stage, the steamed sorghum is extracted and subsequently mixed with 30% water, followed by aeration and cooling processes. Once the temperature reaches 25 °C, Daqu (a microbial fermentation starter) equal to 10% of the initial sorghum weight is added to initiate the first fermentation (referred to as the primary fermentation). In the fifth stage, the entire mixture from the previous step is transferred to a fermentation tank and allowed to ferment for 30 days. In the sixth stage, the fermented material is extracted from the tank and subjected to distillation using a distillation apparatus. In specific terms, 15% of fresh pomegranate peel or rice hull is incorporated into the *Jiupei*, which is then thoroughly mixed and subjected to distillation using a distillation apparatus to produce the base Baijiu. Lastly, a second round of fermentation (referred to as secondary fermentation) and distillation are conducted. This involves fermenting the *Jiupei* obtained from the first distillation to maximize starch utilization. The secondary fermentation process is similar to the primary fermentation, with the exception of adding 7% fresh pomegranate peel to the *Jiupei* [1]. Table 1 presents the detailed experimental design.

#### 3.3.2. Sample Collection

##### Fermented Grain Samples

Fermented grain samples were collected from the fermentation tank on days 0, 10, 20, and 30 of fermentation (with day 0 defined as the initiation day when fermented grains were loaded into the tank), with three replicates performed for each experimental group [39]. Subsequently, the samples were sealed in sterile sampling bags and stored in a −80 °C freezer for subsequent detection and analysis.

##### Light-Flavor Baijiu Samples

The light-flavor Baijiu samples were labeled as L1J, M1J, S1J, and C1J on glass bottles according to their production conditions. Each sample (400 mL per bottle) was stockpiled at 4 °C prior to physicochemical property analysis and gas chromatography–mass spectrometry (GC-MS) detection.

#### 3.3.3. Analysis of Physicochemical Parameters

##### *Jiupei* Samples

During the fermentation process of light-flavor Baijiu, the dry mass, acidity, starch, reducing sugar, and ethanol content in *jiupei* were analyzed according to the method described by You et al. [40]. Briefly, the dry mass was detected using the constant weight method, which involved drying the sample at 105 °C until a constant weight was achieved. The total titratable acidity was quantified via direct titration using a 0.1 M NaOH solution until the pH reached 8.2. Additionally, the levels of reducing sugars and starch were determined via titration.

##### Light-Flavor Baijiu Samples

The levels of solids, total acids, and total esters were measured following established protocols [41]. In brief, the solid content was assessed using a gravimetric approach, where samples were dried at 103 °C until a constant weight was achieved. Total titratable acidity was quantified through direct titration with 0.1 M NaOH solution. Similarly, the total ester content was determined using a titration method involving 0.1 M NaOH and 0.1 M H_2_SO_4_ solutions.

#### 3.3.4. Analysis of the Antioxidant Capacity of Light-Flavor Baijiu

##### Total Phenolic Content

The total phenolic content (TPC) in the samples was detected using the Folin–Ciocalteu method [42]. For each sample, 1 mL of Baijiu was sequentially mixed with 5 mL of Folin–Ciocalteu reagent and 4 mL of Na_2_CO_3_ (75 g/L). The reaction mixture was then diluted with 55 mL of distilled water. After incubation at room temperature for 60 min, the absorbance was measured at a wavelength of 725 nm. A standard curve was prepared using gallic acid (10–400 μg/mL), and the TPC was calculated and expressed as milligram gallic acid equivalents (GAE) per gram of dry weight (dw).

##### Assessment of Antioxidant Properties

The DPPH radical scavenging activity of light-flavor Baijiu samples was evaluated based on a standard protocol with slight modifications [43]. A 0.2 mmol L^−1^ DPPH stock solution was prepared using 80% ethanol and kept in the dark for 3.5 h. Then, 2 mL of the Baijiu sample was mixed with 2 mL of the 0.2 mmol L^−1^ DPPH solution and incubated in the dark for 30 min. The absorbance was gauged at a wavelength of 517 nm. The DPPH scavenging activity was computed using the following formula:

Scavenging rate (%) = (X_0_ − X_1_)/X_0_ × 100

where X_0_ is the absorbance of the control group, and X_1_ is the absorbance of the sample group.

#### 3.3.5. Analysis of Flavor Components

The flavor components in *jiupei* and Baijiu were analyzed using headspace solid-phase microextraction coupled with gas chromatography–mass spectrometry (HS-SPME-GC-MS) [44,45,46]. In specific terms, 1 g of *jiupei* or 1 mL of Baijiu, 5 mL of saturated sodium chloride solution, and 10 μL of 2-octanol (1.0 mg/mL, in methanol of chromatographic purity) were placed in a headspace vial [47,48]. The extraction conditions were as follows: the vial was preequilibrated in a 50 °C water bath for 15 min, followed by adsorption and extraction for 45 min [49]. The fiber was then inserted into the GC injection port, and compounds were desorbed at 250 °C for 5 min. Volatile compounds were separated using a J&W DB-5ms column (30 m × 0.25 mm, 0.25 μm, Agilent Technologies, CA, USA). Helium (99.999%) was used as the carrier gas at a flow rate of 1.6 mL/min in splitless injection mode. The oven temperature program started at 40 °C, held for 1 min, then increased to 80 °C at 10 °C/min, followed by a rise to 140 °C at 3 °C/min, and finally to 220 °C at 20 °C/min, where it was held for 20 min. The mass spectrometer was operated in electron ionization (EI) mode at 70 eV, with the ion source, transfer line, and quadrupole temperatures set at 230 °C, 250 °C, and 150 °C, respectively. Raw data were processed using Agilent GC-MS Analysis software (MassHunter Workstation version 5977C). The mass spectra of flavor compounds were compared with the NIST 17 database for identification. For compounds with authentic standards, retention times were compared under the same conditions to confirm their identities.

##### Quantification of Flavor Compounds

The relative concentration of volatile substances was semi-quantitatively determined using the ratio of the peak area of the internal standard to the peak area of the flavor substance [50,51]. The calculation method is as follows:

$$X_{i}=\frac{{N\times Z}_{i}}{Z_{s}}\cdot X_{s}$$

where X_i_ represents the diluted mass concentration of the flavor substance (mg/L); Xs represents the final concentration of the internal standard solution (mg/L); Z_i_ and Zs are the peak areas of the flavor substance and the internal standard solution, respectively; and N is the dilution factor.

#### 3.3.6. Statistical Analysis

Three replicates were performed for each experimental group, and the results were averaged. One-way analysis of variance (ANOVA) was performed using GraphPad Prism 9.5 software (GraphPad Software, Inc., Boston, MA, USA), followed by post hoc comparisons using Tukey’s test. Considerable differences were determined using Duncan’s multiple range test with a prominence threshold of *p* < 0.05. Pearson correlation coefficient was used to assess the relationships between the physical and chemical properties of *jiupei*. Graphs and charts were generated using Origin (2024) and IBM SPSS Statistics 27. Data are presented as mean ± standard deviation (SD).

## 4. Conclusions

The dry mass of *jiupei* exhibited a substantial negative association with reducing sugar content (*p* < 0.01) while showing substantial positive correlations with acidity (*p* < 0.01) and alcohol content (*p* < 0.01). Additionally, a positive association was observed between acidity and flavor compounds (*p* < 0.001). Fresh pomegranate peels of medium size (1.5 × 0.3 × 0.3 cm^3^) were more effective than rice hulls in reducing furfural content in Baijiu (from 9.00 ± 0.21 mg/L to 0.90 ± 0.15 mg/L) and enhancing the antioxidant capacity of Baijiu. They also contributed to the production of linalool (52.44 ± 2.50 mg/L), which added flavor to the Baijiu. Furthermore, this Baijiu meets the standards for high-quality Baijiu as defined by the Chinese National Standard for light-flavor Baijiu [21]. Notably, when the pomegranate peels were crushed to a size of 1.5 × 0.3 × 0.3 cm^3^, the total yield of Baijiu reached its highest level at 48% ± 3.41%.

This study confirms the feasibility of incorporating fresh pomegranate peels as an auxiliary material in the yield of light-flavor Baijiu. Substituting rice hulls with medium-sized (1.5 × 0.3 × 0.3 cm^3^) pomegranate peels at 15% of the raw material weight improved the quality of Baijiu, enhanced its flavor, and increased its antioxidant capacity. The findings not only provide a theoretical foundation for using fresh pomegranate peels or rice hulls in brewing light-flavor Baijiu (or other flavor types of Baijiu) but also offer insights into new applications for pomegranate peels.

## Figures and Tables

**Table 1:** Pomegranate peel experimental group subjected to two sequential fermentation batches.

Ams ^1^	Granular Dimension (Length (cm) × Width (cm) × Height (cm))	First Fermentation			Second Fermentation		
		*Jiupei* No.	AMs Added Amount (%)	Baijiu No.	*Jiupei* No.	AMs Added Amount (%)	Baijiu No.
GFPP	2.5 × 0.3 × 0.3	L1	15%	L1J	L2	7%	L2J
GFPP	1.5 × 0.3 × 0.3	M1	15%	M1J	M2	7%	M2J
GFPP	0.5 × 0.3 × 0.3	S1	15%	S1J	S2	7%	S2J
RH	0.8 × 0.33 × 0.28	C1	15%	C1J	C2	7%	C2J

^1^ Abbreviations: AM, auxiliary material; GFPP, granular-textured fresh pomegranate peel; RH, rice hull.

## Data Availability

The data have been fully provided.

## Supplementary Materials

The following supporting information can be downloaded at: https://www.mdpi.com/article/10.3390/molecules30081800/s1, Figure S1: Types (a,b) and contents (c,d) of volatile flavor compounds in light-flavor base Baijiu. The colors of the compounds were as follows: others (brown), ethers (purplish red), phenols (purple), ketones (blue), aldehydes (green), acids (yellow), alcohols (light red), and esters (orange). Table S1: Esters in light-flavor base Baijiu (first fermentation) [3,52]. Table S2: Alcohols in light-flavor base Baijiu (first fermentation) [3,52]. Table S3: Acids, aldehydes, and ketones in light-flavor base Baijiu (first fermentation) [3,52]. Table S4: Other compounds in light-flavor base Baijiu (first fermentation) [3,52]. Table S5: Esters in light-flavor base Baijiu (second fermentation) [3,52]. Table S6: Alcohols in light-flavor base Baijiu (second fermentation) [3,52]. Table S7: Acids, aldehydes, and ketones in light-flavor base Baijiu (second fermentation) [3,52]. Table S8: Other compounds in light-flavor base Baijiu (second fermentation) [3,52]. Table S9: Volatile flavor compounds in *Jiupei* (first fermentation). Table S10: Volatile flavor compounds in *Jiupei* (second fermentation).
