# Supplementary material for: Pomegranate Peel as a Sustainable Additive for Baijiu Fermentation: Physicochemical and Flavor Analysis with Process Optimization
Source: Molecules. 2025 Apr 17;30(8):1800. doi: 10.3390/molecules30081800 (PMC12029601; doi:10.3390/molecules30081800)
Supplement: Supplementary file 1 [file molecules-30-01800-s001.zip › molecules-3550993-supplementary.pdf]

Supplemental Information

Pomegranate Peel as a Sustainable Additive for Baijiu Fermentation: Physicochemical and Flavor Analysis with Process Optimization

Longwen Wang [1], Guida Zhu [1], Na Li [2], Zhiheng Wang [1], Yi Ji [1], Chen Shen [3], Jing Yu [1,*] and Ping Song [1,*]

[1] School of Food Science and Pharmaceutical Engineering, Nanjing Normal University, Nanjing 210023, China; 232712052@njnu.edu.cn (L.W.); 232702011@njnu.edu.cn (G.Z.); 232712046@njnu.edu.cn (Z.W.); 28230130@njnu.edu.cn (Y.J.)

[2] Huaguan Group Brewery Co., Ltd., Heze 274900, China; 15020470277@163.com

[3] Shimadzu (China) Co., Ltd., Shanghai 200233, China; sshshenc@shimadzu.com.cn

* Correspondence: jingyu@njnu.edu.cn (J.Y.); songping@njnu.edu.cn (P.S.)

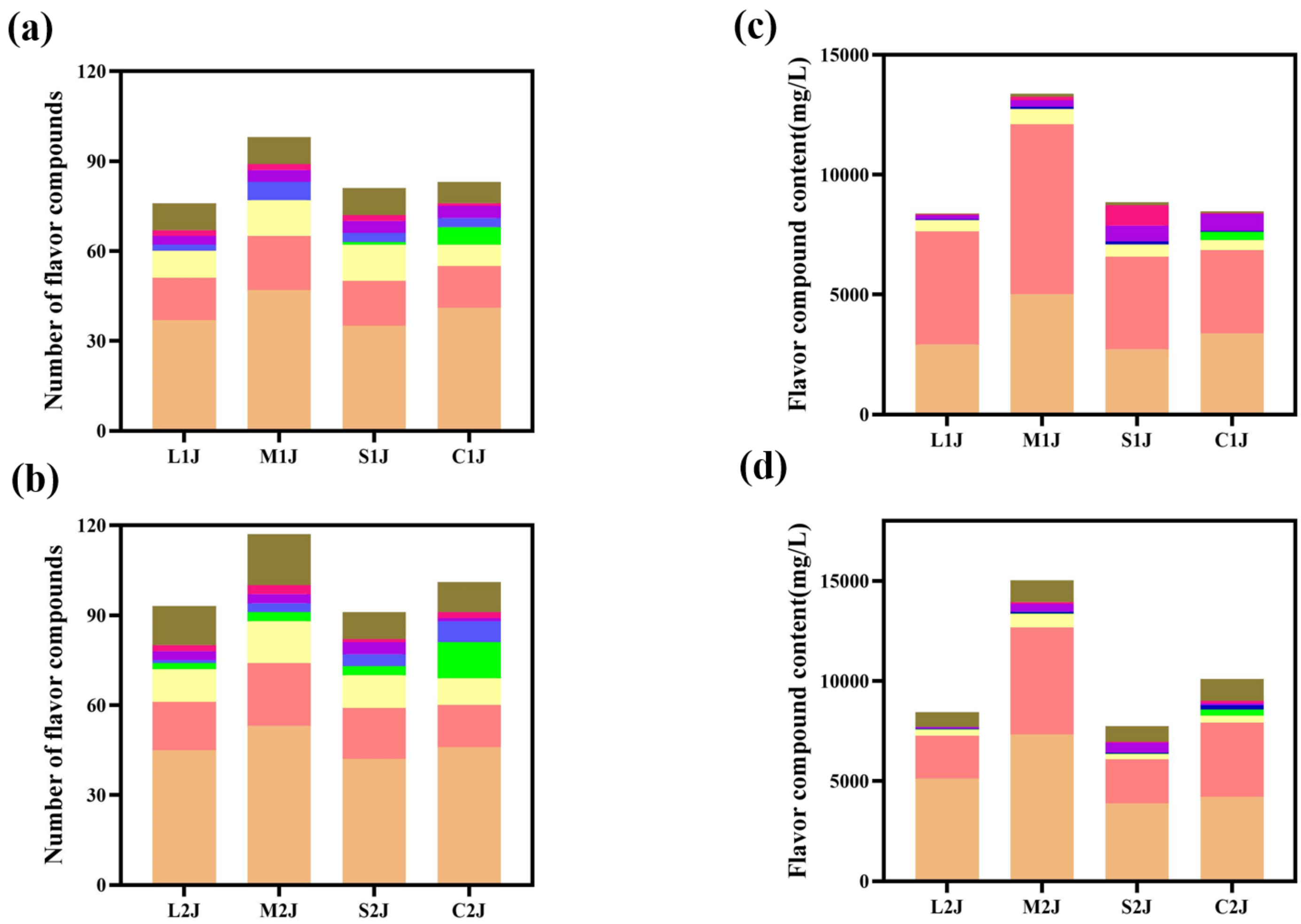

Figure S1. Types (a), (b) and contents (c), (d) of volatile flavour compounds in light-flavor base Baijiu. The colours of the compounds were as follows: others (brown), ethers (purplish red), phenols (purple), ketones (blue), aldehydes (green), acids (yellow), alcohols (light red) and esters (orange).

Table S1. Esters in light-flavor base Baijiu (first fermentation) [3,52]

name	Sensation threshold (mg/L)	L1J (mg/L)	M1J (mg/L)	S1J (mg/L)	C1J (mg/L)
Ethyl Acetate	32. 55	373.53±21.23	293.86±28.24	496.78±22.41	369.75±33.15
Boronic acid, ethyl-, diethyl ester	-	0.00±0.00	0.33±0.04	0.00±0.00	0.00±0.00
Methylazoxymethanol acetate	-	26.47±1.26	1.75±0.21	0.00±0.00	0.00±0.00
Butanoic acid, ethyl ester	0.082	630.91±42.16	1428.29±88.26	614.07±31.05	498.06±24.89
Acetic acid, butyl ester	-	0.00±0.00	362.65±17.54	0.00±0.00	0.00±0.00
1-Butanol, 3-methyl-, acetate	0.094	11.65±1.07	22.77±4.55	8.78±1.24	72.65±14.70
Pentanoic acid, ethyl ester	0.027	1.87±0.32	3.13±0.78	1.44±0.21	3.02±0.017
Butanoic acid, 2-methylpropyl ester	-	2.31±0.25	19.60±3.47	0.00±0.00	0.00±0.00
Butanoic acid, butyl ester	14.07	72.79±7.58	228.65±35.86	0.00±0.00	5.68±1.26
Hexanoic acid, ethyl ester	0.055	35.73±2.89	33.57±3.14	96.83±5.76	134.86±9.86
Butanoic acid, 3-methylbutyl ester	0.015	0.00±0.00	35.22±5.42	0.00±0.00	0.00±0.00
Propanoic acid, 2-hydroxy-, ethyl ester	128.08	20.49±2.43	4.14±0.73	15.01±1.99	34.39±3.43
Heptanoic acid, ethyl ester	13.15	0.00±0.00	3.70±0.73	3.97±0.82	21.22±2.67
Octanoic acid, ethyl ester	0.013	27.26±3.06	25.37±2.43	22.70±2.57	127.61±12.47
Ethyl dl-2-hydroxycaproate	-	0.00±0.00	3.34±0.72	0.00±0.00	0.00±0.00
Nonanoic acid, ethyl ester	3.15	4.90±0.92	9.22±1.11	3.34±0.68	29.01±3.35
Benzoic acid, ethyl ester	1.43	5.98±1.32	8.80±2.04	3.30±0.75	0.00±0.00
Decanoic acid, ethyl ester	1.12	9.68±2.42	10.44±3.74	6.33±1.55	30.06±4.40
Butanedioic acid, diethyl ester	353.19	10.90±2.17	36.89±4.15	0.00±0.00	0.00±0.00
Benzeneacetic acid, ethyl ester	0.407	6.71±1.51	6.85±1.62	2.65±0.37	10.38±2.07
Acetic acid, 2-phenylethyl ester	0.91	6.29±1.44	9.20±2.12	5.90±1.00	28.69±4.15
Succinic acid, butyl ethyl ester	-	0.00±0.00	7.38±1.13	0.00±0.00	0.00±0.00
2(3H)-Furanone, dihydro-5-pentyl	-	4.07±0.88	10.03±2.47	11.70±3.54	0.00±0.00
Tetradecanoic acid, ethyl ester	-	13.68±2.46	37.20±5.61	0.00±0.00	0.00±0.00
(E)-2-Propenoic acid, 3-phenyl-, ethyl ester	-	0.00±0.00	9.14±2.04	0.00±0.00	21.29±3.42
Diethyl suberate	-	19.47±2.72	26.29±4.10	22.27±3.21	27.69±4.13
Pentadecanoic acid, ethyl ester	-	4.47±0.77	23.45±3.55	0.00±0.00	9.09±1.85
Diethyl azelate	-	61.68±8.78	107.11±10.14	52.40±5.67	45.86±7.20
Dimethyl phthalate	-	5.81±1.14	10.69±1.89	5.44±1.03	6.27±1.24

Hexadecanoic acid, ethyl ester	14	579.44±77.57	1119.59±102.41	519.14±66.74	765.14±80.59
Ethyl 9-hexadecenoate	-	34.25±6.36	98.15±10.14	23.06±4.05	63.71±9.79
Decanedioic acid, diethyl ester	-	0.00±0.00	2.08±0.42	0.00±0.00	0.00±0.00
Heptadecanoic acid, ethyl ester	-	2.15±0.41	5.03±1.62	0.00±0.00	3.14±0.75
10-Undecenoic acid, ethyl ester	-	0.00±0.00	1.10±0.15	0.00±0.00	0.00±0.00
Octadecanoic acid, ethyl ester	-	18.22±2.58	30.48±5.65	11.49±2.37	21.31±4.85
(E)-9-Octadecenoic acid ethyl ester	-	275.01±22.47	382.63±33.17	240.30±25.42	353.51±37.49
9,12-Octadecadienoic acid (Z,Z)-, 2,3-dihydroxypropyl ester	-	0.00±0.00	2.79±0.34	0.00±0.00	0.00±0.00
Methyl .gamma.-linolenate	-	0.00±0.00	11.00±2.42	0.00±0.00	0.00±0.00
9,12,15-Octadecatrienoic acid, methyl ester	-	4.32±0.56	30.80±4.72	0.00±0.00	20.85±3.41
Linoleic acid ethyl ester	0.45	340.21±40.52	521.45±61.82	336.85±42.77	414.34±53.33
9,12,15-Octadecatrienoic acid, ethyl ester	-	0.00±0.00	5.57±0.64	0.00±0.00	0.00±0.00
Dibutyl phthalate	-	4.32±0.58	6.26±0.73	4.07±0.59	4.89±
Dodecanoic acid, ethyl ester	-	5.32±0.61	10.15±2.52	2.73±0.34	0.00±0.00
.beta.-Phenylethyl butyrate	-	6.29±0.77	15.39±3.62	0.00±0.00	0.00±0.00
Pentanoic acid, 3-acetyl-4-oxo-, ethyl ester	-	0.00±0.00	1.69±0.27	3.53±0.45	6.72±0.78
Tetradecanoic acid, ethyl ester	-	0.00±0.00	0.00±0.00	0.00±0.00	29.61±4.72
(S)-Isopropyl lactate	-	39.25±5.98	0.00±0.00	0.00±0.00	0.00±0.00

Table S2. Alcohols in light-flavor base Baijiu (first fermentation) [3,52]

Name	Sensation threshold (mg/L)	L1J (mg/L)	M1J (mg/L)	S1J (mg/L)	C1J (mg/L)
Ethanol	14000	4404.41±550.75	6277.87±752.50	3358.51±428.63	2828.03±377.18
2-Butanol, 3-methyl	-	0.00±0.00	0.95±0.07	0.00±0.00	0.00±0.00
1-Propanol, 2-methyl	40	20.83±3.79	53.26±6.62	4.49±0.56	28.99±3.54
1-Butanol	2.73	36.97±4.95	26.20±3.36	3.63±0.47	230.12±32.21
1-Butanol, 2-methyl	2.50	25.51±3.81	74.56±8.12	14.04±2.11	0.00±0.00
Linalool	-	40.53±5.27	52.44±2.50	41.03±5.08	0.00±0.00
1-Butanol, 3-methyl	179.19	126.11±13.24	357.33±36.41	142.76±15.43	231.30±24.85
1-Hexanol	5.37	7.27±0.88	19.91±2.03	8.60±0.99	12.58±2.71
3-Octanol	-	0.00±0.00	2.80±0.34	0.00±0.00	0.00±0.00
1-Octen-3-ol	-	0.00±0.00	9.91±1.07	0.00±0.00	0.00±0.00
1-Heptanol	2.50	2.79±0.38	17.19±1.80	0.00±0.00	4.36±0.58
1-Hexanol, 2-ethyl	0.27	4.40±0.52	11.07±2.13	0.00±0.00	5.84±0.68
2,3-Butanediol, [R-(R*,R*)]	150	5.39±0.69	7.23±0.84	5.94±0.68	12.70±2.35
1-Nonanol	0.12	10.64±2.10	43.83±5.72	16.26±2.41	0.00±0.00
1-Decanol	0.40	0.00±0.00	5.78±0.67	0.00±0.00	2.78±0.35
5-Decen-1-ol, (E)-	-	0.00±0.00	6.47±0.72	0.00±0.00	0.00±0.00
Phenylethyl Alcohol	28.92	20.93±3.43	53.75±6.84	19.93±2.87	29.78±3.91
1-Octanol	1.10	5.63±0.68	58.71±6.18	5.95±0.69	15.49±2.28

Table S3. Acids, aldehydes, and ketones in light-flavor base Baijiu (first fermentation) [3,52]

Name	Sensation threshold (mg/L)	L1J (mg/L)	M1J (mg/L)	S1J (mg/L)	C1J (mg/L)
n-Hexadecanoic acid	-	4.58±0.57	5.90±0.64	4.02±0.52	0.00±0.00
Dodecanoic acid	-	0.00±0.00	10.49±2.02	5.52±0.63	7.10±0.81
trans-2-Decenoic acid	-	0.00±0.00	5.53±0.67	0.00±0.00	0.00±0.00
2-Oxopentanedioic acid	-	0.00±0.00	3.17±0.47	0.00±0.00	0.00±0.00
Acetic acid	160	82.27±9.97	176.16±18.73	139.41±14.85	53.06±6.97
Propanoic acid, 2-methyl	1.58	0.00±0.00	9.90±1.24	0.00±0.00	0.00±0.00
Butanoic acid	0.96	199.31±20.51	89.55±9.33	124.81±13.47	221.37±23.83
Hexanoic acid	2.52	82.82±9.24	60.24±7.14	54.63±6.74	95.62±10.82
Heptanoic acid	13.82	0.00±0.00	8.38±0.91	0.00±0.00	0.00±0.00
Octanoic acid	2.70	35.38±3.60	83.46±8.47	29.13±3.12	22.01±3.17
Nonanoic acid	3.56	29.76±4.25	126.59±13.34	24.18±3.42	22.08±2.88
n-Decanoic acid	13.74	33.39±3.79	57.10±6.14	39.18±4.28	0.00±0.00
2-Nonanone	-	0.00±0.0	6.99±0.76	0.00±0.00	0.00±0.00
5,9-Undecadien-2-one, 6,10-dimethyl	-	0.45±0.05	10.05±1.89	1.22±0.26	0.00±0.00
(Z)-Oxacyclopentadec-6-en-2-one	-	0.00±0.00	10.84±2.16	0.00±0.00	7.49±0.84
3-Octanone	-	0.00±0.00	8.82±0.92	0.00±0.00	0.00±0.00
Acetoin	0.26	38.92±4.12	44.16±5.25	30.82±4.55	19.63±2.67
Ethane, 1,1-diethoxy	0.05	0.00±0.00	0.00±0.00	0.00±0.00	40.30±5.82
Hexanal	0.025	0.00±0.00	0.00±0.00	0.00±0.00	253.00±34.05
Furfural	-	1.50±0.28	0.90±0.15	1.00±0.22	9.00±1.21
2-Undecenal	-	0.00±0.00	0.00±0.00	0.00±0.00	12.27±2.52
2,4-Decadienal, (E,E)	-	0.00±0.00	0.00±0.00	0.00±0.00	13.82±2.02
2(3H)-Furanone, dihydro-5-pentyl	-	0.00±0.00	0.00±0.00	0.00±0.00	17.61±2.67

Table S4. Other compounds in light-flavor base Baijiu (first fermentation) [3,52]

Name	Sensation threshold (mg/L)	L1J (mg/L)	M1J (mg/L)	S1J (mg/L)	C1J (mg/L)
Dimethyl ether	-	30.11±4.21	106.98±20.42	772.45±83.63	48.51±5.37
Propane, 1-(1-methylethoxy)	-	0.00±0.00	38.01±4.77	0.00±0.00	0.00±0.00
Phenol	18.91	3.55±0.49	6.27±0.78	2.29±0.35	7.63±0.81
Phenol, 4-ethyl-2-methoxy	-	145.57±15.80	256.85±26.14	648.87±66.92	645.57±66.50
Phenol, 4-ethyl	0.62	13.61±2.64	13.07±2.14	8.90±0.98	52.48±6.45
2-Methoxy-4-vinylphenol	-	0.00±0.00	7.87±0.86	12.72±2.73	5.62±0.68
Ethane, 1-ethoxy-1-methoxy	-	0.00±0.00	1.02±0.24	0.45±0.07	0.00±0.00
Butane, 1,1-diethoxy-3-methyl	3.00	0.00±0.00	79.31±8.11	0.00±0.00	0.00±0.00
1,3-Dioxepane, 2-pentadecyl	-	0.00±0.00	2.86±3.82	0.00±0.00	0.00±0.00
Cyclononasiloxane, octadecamethyl	-	1.78±0.26	1.66±0.29	2.50±0.32	6.95±0.77
Cyclohexanamine, N-(benzoyloxy)	-	0.00±0.00	16.27±2.63	0.00±0.00	0.00±0.00
Pyridine, 2,6-diamino-3-((2,5-dichloropenyl)azo)	-	0.00±0.00	3.25±0.45	0.00±0.00	0.00±0.00
3,4-Dihydroxyphenylglycol, 4TMS derivative	-	1.33±0.23	1.31±0.20	0.00±0.00	2.37±0.34
Benzofuran, 2,3-dihydro	-	4.39±0.58	4.49±0.56	0.00±0.00	0.00±0.00
3,4-Dihydroxymandelic acid, 4TMS derivative	-	0.00±0.00	1.04±0.27	0.00±0.00	0.00±0.00
Cyclopentasiloxane, decamethyl	-	0.00±0.00	0.00±0.00	0.00±0.00	8.01±0.99
Methane, nitroso	-	3.66±0.43	0.00±0.00	0.00±0.00	0.21±0.03
Hexasiloxane, 1,1,3,3,5,5,7,7,9,9,11,11-dodecamethyl	-	1.11±0.28	0.00±0.00	1.84±0.26	3.36±0.46
Cyclohexasiloxane, dodecamethyl	-	0.00±0.00	0.00±0.00	0.00±0.00	3.61±0.47
Tritetracontane	-	3.25±0.46	0.00±0.00	0.00±0.00	0.00±0.00
Hexadecane, 1,1-bis(dodecyloxy)	-	0.00±0.00	0.00±0.00	3.55±0.48	0.00±0.00
Cyclohexasiloxane, dodecamethyl	-	0.00±0.00	0.00±0.00	1.69±0.25	0.00±0.00

Table S5. Esters in light-flavor base Baijiu (second fermentation) [3,52]

Name	Sensation threshold (mg/L)	L2J (mg/L)	M2J (mg/L)	S2J (mg/L)	C2J (mg/L)
Ethyl Acetate	32.56	648.31±75.12	1494.77±150.86	543.75±63.42	540.51±62.01
Butanoic acid, ethyl ester	0.082	1618.53±245.55	3198.31±423.57	896.97±90.18	1252.10±130.86
Butyl lactate	-	0.00±0.00	8.59±0.98	0.00±0.00	0.00±0.00
Acetic acid, butyl ester	-	21.88±3.14	27.34±3.25	0.00±0.00	25.41±3.38
Propanoic acid, 2-hydroxy-, ethyl ester	-	5.60± 0.69	0.00±0.00	7.42±0.84	8.57±0.97
1-Butanol, 3-methyl-, acetate	0.094	8.58±0.95	62.75±7.72	5.58±0.65	22.58±3.55
Pentanoic acid, ethyl ester	0.027	2.10±0.36	15.34±2.48	1.09±0.27	4.66±0.55
2-Butenoic acid, ethyl ester	-	1.73±0.29	1.02±0.22	1.76±0.27	0.00±0.00
Butanoic acid, 2-methylpropyl ester	-	1.76±0.28	10.11±2.89	2.91±0.35	0.00±0.00
Pentanoic acid, 4-methyl-, ethyl ester	0.005	1.10±0.21	1.37±0.27	0.00±0.00	0.00±0.00
Butanoic acid, butyl ester	14.07	72.46±8.94	140.70±15.61	40.70±5.57	0.00±0.00
Hexanoic acid, ethyl ester	0.055	156.06±16.17	302.98±31.52	126.71±13.18	70.25±8.99
Butanoic acid, 3-methylbutyl ester	0.015	6.50±0.73	64.61±7.05	4.19±0.58	36.11±4.18
Heptanoic acid, ethyl ester	13.15	77.20±8.73	124.62±13.45	77.62±8.55	5.96±0.69
Octanoic acid, ethyl ester	0.013	440.06±55.21	776.30±82.20	315.01±40.54	40.66±48.73
Hexanoic acid, butyl ester	0.68	1.03±0.25	1.42±0.23	0.00±0.00	0.00±0.00
Butanoic acid, heptyl ester	-	0.00±0.00	0.00±0.00	0.00±0.00	216.85±32.57
8-Methylnonanoic acid, ethyl ester	-	5.89±0.66	7.68±0.89	13.82±2.14	43.32±5.52
Ethyl dl-2-hydroxycaproate	-	0.00±0.00	7.37±0.86	0.00±0.00	5.62±0.66
Nonanoic acid, ethyl ester	3.15	108.69±11.72	308.66±42.25	99.02±10.62	12.84±2.67
Ethyl (E)-2-octenoate	-	0.00±0.00	2.33±0.38	0.00±0.00	0.00±0.00
3-Nonenoic acid, ethyl ester	-	0.00±0.00	11.60±2.05	0.00±0.00	0.00±0.00
Decanoic acid, ethyl ester	1.12	329.43±41.17	660.09±70.43	210.09±30.16	17.52±2.82
Ethyl trans-4-decenoate	0.11	0.00±0.00	12.86±2.45	0.00±0.00	0.00±0.00
Octanoic acid, 3-methylbutyl ester	0.13	0.00±0.00	4.47±0.58	0.00±0.00	0.00±0.00
Ethyl 9-decenoate	-	0.00±0.00	26.09±3.31	0.00±0.00	0.00±0.00
Benzeneacetic acid, ethyl ester	0.41	6.83±0.74	11.73±2.75	6.33±0.73	9.83±1.05
Undecanoic acid, ethyl ester	-	0.00±0.00	13.78±2.71	0.00±0.00	0.00±0.00
Ethyl trans-2-decenoate	-	0.00±0.00	4.69±0.56	0.00±0.00	0.00±0.00

Acetic acid, 2-phenylethyl ester	0.91	5.14±0.66	14.23±2.52	6.47±0.79	10.84±2.10
Dodecanoic acid, ethyl ester	0.40	100.84±11.58	205.09±25.17	64.16±7.32	10.48±2.78
.beta.-Phenylethyl butyrate	-	3.38±0.44	19.52±2.82	3.52±0.48	15.11±2.89
Formic acid, octyl ester	-	0.00±0.00	0.00±0.00	3.39± 0.47	14.17±2.20
Ethyl tridecanoate	-	0.00±0.00	4.49±0.58	0.00±0.00	0.00±0.00
Tetradecanoic acid, ethyl ester	0.50	4.90±0.54	254.32±30.72	0.00±0.00	6.31±0.76
2-Ethylbutyric acid, hexyl ester	-	0.00±0.00	4.69±0.58	0.00±0.00	0.00±0.00
Diethyl suberate	-	14.36±2.14	24.24±3.62	9.84±1.88	17.33±2.45
Linoleyl acetate	-	0.00±0.00	10.96±2.12	0.00±0.00	0.00±0.00
Pentadecanoic acid, ethyl ester	-	0.00±0.00	81.45±9.34	8.08±1.07	8.17±1.15
Ethyl 13-methyl-tetradecanoate	-	0.00±0.00	63.06 ±7.56	0.00±0.00	0.00±0.00
Diethyl azelate	-	50.41±6.35	61.58±7.40	27.26±3.80	69.05±8.90
Hexadecanoic acid, ethyl ester	14	749.52±80.12	1319.36±201.03	630.84±71.43	840.48±92.05
Methyl hexadec-9-enoate	-	0.00±0.00	30.33±4.54	0.00±0.00	0.00±0.00
Ethyl 9-hexadecenoate	-	103.39±15.42	217.23±22.30	122.19±14.72	54.46±60.10
Decanedioic acid, diethyl ester	-	0.00±0.00	4.37±0.55	0.00±0.00	1.40±0.20
n-Propyl 9,12-hexadecadienoate	-	0.00±0.00	9.34±1.04	0.00±0.00	0.00±0.00
Heptadecanoic acid, ethyl ester	-	0.00±0.00	2.38±0.33	3.61±0.45	3.61±0.48
(Z)-Ethyl heptadec-9-enoate	-	0.00±0.00	1.66±0.28	0.00±0.00	0.00±0.00
Octadecanoic acid, ethyl ester	-	6.74±0.75	6.41±0.77	5.91±0.72	22.38±3.43
(E)-9-Octadecenoic acid ethyl ester	-	101.64±14.05	231.25±32.25	261.11±34.18	285.49±33.39
Linoleic acid ethyl ester	0.45	342.64±38.90	408.79±52.12	302.89±38.90	344.41±42.08
Propanoic acid, 2-methyl-, butyl ester	-	29.21±3.86	0.00±0.00	0.00±0.00	0.00±0.00
9,12,15-Octadecatrienoic acid, methyl ester	-	6.25±0.75	20.38±3.23	3.40±0.45	16.02±2.86
Acetic acid, ethoxyhydroxy-, ethyl ester	-	2.51±0.39	3.98±0.41	0.00±0.00	0.00±0.00
Cyclopentanecarboxylic acid, 3-methylbutyl ester	-	5.03±0.68	7.48±0.83	0.00±0.00	0.00±0.00
Nonanoic acid, 9-oxo-, ethyl ester	-	0.00±0.00	0.00±0.00	12.69±2.05	29.26±3.45
Cyclopentanecarboxylic acid, 3-methylene-2-methyl-4-pentyl-, methyl ester	-	0.00±0.00	4.05±0.58	0.00±0.00	0.00±0.00
cis,cis,cis-6,9,12-Octadecatrienoic acid, propyl ester	-	0.00±0.00	6.11±0.77	0.00±0.00	0.00±0.00

Table S6. Alcohols in light-flavor base Baijiu (second fermentation) [3,52]

Name	Sensation threshold (mg/L)	L2J (mg/L)	M2J (mg/L)	S2J (mg/L)	C2J (mg/L)
Ethanol	14	1912.00±201.05	5297.68±627.72	1788.88±210.48	3100.24±382.07
1-Butanol	2.73	29.88±3.89	29.51±3.75	3.96±0.47	2.05±0.31
1-Propanol, 2-methyl	40	1.54±0.22	43.33±5.17	0.00±0.00	11.07±2.12
1-Butanol, 2-methyl	65	17.24±2.12	42.45±5.24	11.43±2.75	40.66±5.55
Linalool	-	26.41±3.46	31.27±4.88	23.99±3.76	0.00±0.00
2-Butanol	-	0.00±0.00	0.00±0.00	2.26±0.37	196.23±25.73
1-Butanol, 3-methyl	179.19	69.40±7.83	393.97±48.73	61.36±7.08	209.33±28.46
1-Hexanol	5.37	4.78±0.57	23.06±3.05	5.07±0.69	14.18±2.52
1-Heptanol	2.50	18.32±2.47	22.71±3.58	15.86±2.01	3.59± 0.46
Ethanol, 2-nitro	-	0.00±0.00	238.27±33.52	225.52±28.19	0.00±0.00
1-Nonanol	0.12	13.58±2.05	56.92±6.20	14.37±2.25	25.76±3.12
1-Octen-3-ol	-	3.11±0.41	4.96±0.58	3.57±0.46	0.00±0.00
1-Hexanol, 2-ethyl	0.27	0.00±0.00	3.73±0.47	1.11±0.22	0.00±0.00
1-Octanol	1.10	3.76±0.45	54.10±6.68	3.38±0.41	15.61±2.25
1-Decanol	0.40	0.00±0.00	6.40±0.72	0.00±0.00	0.00±0.00
2-Butanol, 3-chloro	-	0.00±0.00	0.00±0.00	0.00±0.00	47.01±6.21
2-Butanol, 3-methyl	-	4.75±0.59	0.00±0.00	0.00±0.000	0.00±0.00
Phenylethyl Alcohol	28.92	22.32±3.30	56.33±6.68	23.05±3.47	34.57±4.76
4-Heptanol, 2,6-dimethyl-	-	0.00±0.00	0.00±0.00	0.00±0.00	2.52±0.38
1,6,10-Dodecatrien-3-ol, 3,7,11-trimethyl	-	0.00±0.00	10.31±2.15	0.00±0.00	0.00±0.00
2,3-Butanediol	150.00	5.03±0.62	4.82±0.53	2.97±0.37	0.00±0.00
4-Methoxycarbonyl-4-butanolide	-	0.00±0.00	4.04±0.55	4.25±0.50	4.58±0.57
trans-Farnesol	-	0.00±0.00	9.93±1.73	0.00±0.00	0.00±0.00
(E)-5-Decen-1-ol	-	0.00±0.00	9.02±2.05	0.00±0.00	0.00±0.00
2,6,10-Dodecatrien-1-ol, 3,7,11-trimethyl-	-	0.00±0.00	7.95±0.83	0.00±0.00	0.00±0.00

Table S7. Acids, aldehydes, and ketones in light-flavor base Baijiu (second fermentation) [3,52]

Name	Sensation threshold (mg/L)	L2J (mg/L)	M2J (mg/L)	S2J (mg/L)	C2J (mg/L)
Nonanoic acid	3.56	16.11±2.03	103.44±11.55	17.86±2.14	34.24±4.65
8-Methylnonanoic acid	-	34.22±4.18	93.83±10.20	24.10±3.52	0.00±0.00
Tetradecanoic acid	-	1.88±0.28	2.25±0.34	2.25±0.38	0.00±0.00
n-Hexadecanoic acid	-	0.00±0.00	7.02±0.83	0.00±0.00	10.12±2.14
Dodecanoic acid	-	2.53±0.38	16.05±2.41	0.00±0.00	5.60±0.68
n-Decanoic acid	13.74	24.93±3.17	76.28±8.15	21.78±3.15	49.09±5.90
L-Lactic acid	-	50.87±6.52	74.52±8.72	42.10±5.42	0.00±0.00
Heptanoic acid	13.82	0.00±0.00	10.33±2.50	0.00±0.00	0.00±0.00
Acetic acid	160	90.08±10.40	136.19±14.53	56.46±6.54	89.69±9.79
Formic acid	-	0.00±0.00	0.00±0.00	0.00±0.00	83.66±9.89
Butanoic acid	0.96	37.00±4.70	168.14±20.18	49.73±5.80	22.70±3.42
Hexanoic acid	2.52	22.33±3.15	18.71±2.25	18.65±2.43	24.05±3.48
Octanoic acid	2.70	21.39±3.70	58.33±6.82	23.99±3.18	36.54±4.15
Arachidonic acid	-	0.00±0.00	13.11±2.71	0.00±0.00	0.00±0.00
Hexanal	25.48	0.00±0.00	0.00±0.00	0.00±0.00	14.44±2.28
Paraldehyde	-	0.37±0.04	1.93±0.29	0.00±0.00	0.00±0.00
2-Undecenal	-	0.00±0.00	0.00±0.00	0.00±0.00	14.45±2.73
2,4-Decadienal	-	0.00±0.00	0.00±0.00	0.00±0.00	11.44±2.82
Ethane, 1,1-diethoxy	50	0.00±0.00	0.00±0.00	0.00±0.00	191.27±25.74
Octane, 1,1-diethoxy	-	0.00±0.00	0.00±0.00	0.00±0.00	20.76±3.08
8-Hexadecenal, 14-methyl	-	0.00±0.00	2.61±0.34	0.00±0.00	0.00±0.00
2(3H)-Furanone, dihydro-5-pentyl	-	14.22±2.27	14.84±2.58	14.09±2.80	15.84±2.01
Acetoin	0.259	27.40±3.51	94.87±10.73	40.60±5.27	153.06±18.74
3-Octanone	21	0.00±0.00	0.00±0.00	5.60±0.68	5.48±0.64
2-Nonanone	483	0.00±0.00	0.00±0.00	2.25±0.39	3.14±0.47
2,4(1H,3H)-Pyrimidinedione, dihydro-5,6-dihydroxy-5-methyl	-	0.00±0.00	0.14±0.02	0.00±0.00	0.00±0.00
12-Methyl-oxa-cyclododec-6-en-2-one	-	0.00±0.00	4.12±0.55	0.00±0.00	0.00±0.00
(Z)-Oxacyclopentadec-6-en-2-one	-	0.00±0.00	0.00±0.00	0.00±0.00	34.30±4.27
2-Hexenoic acid, 5-hydroxy-3,4,4-trimethyl	-	0.00±0.00	3.30±0.49	0.00±0.00	0.00±0.00

Table S8. Other compounds in light-flavor base Baijiu (second fermentation) [3,52]

Name	Sensation threshold (mg/L)	L2J (mg/L)	M2J (mg/L)	S2J (mg/L)	C2J (mg/L)
Phenol, 4-ethyl-2-methoxy	0.12	84.34±9.89	342.70±41.24	457.60±50.73	92.34±10.96
Phenol	18.91	0.00±0.00	4.26±0.57	0.00±0.00	0.00±0.00
Phenol, 4-ethyl	0.62	4.15±0.58	25.97±3.76	4.29±0.52	0.00±0.00
Dimethyl ether	-	3.82±0.46	104.79±15.62	50.44±6.41	120.99±18.73
Octaethylene glycol monododecyl ether	-	0.00±0.00	0.26±0.03	0.00±0.00	0.48±0.05
Cyclotetrasiloxane, octamethyl-	-	1.52±0.27	0.00±0.00	1.20±0.23	0.00±0.00
Ethane, 1-ethoxy-1-methoxy	-	3.55±0.49	2.88±0.37	0.00±0.00	0.00±0.00
Cyclohexanamine, N-(benzoyloxy)	-	0.00±0.00	0.00±0.00	0.00±0.00	26.15±3.73
1,3-Bis(cinnamoyloxymethyl)adamantane	-	113.43±17.82	133.71±23.42	0.00±0.00	0.00±0.00
Tetratetracontane	-	2.72±0.35	4.45±0.50	0.00±0.00	0.00±0.00
Butane, 1,1-diethoxy-3-methyl	3.00	0.00±0.00	14.74±2.51	0.00±0.00	0.00±0.00
Benzene, (2,2-diethoxyethyl)	-	0.00±0.00	0.00±0.00	0.00±0.00	24.00±3.81
Cyclohexasiloxane, dodecamethyl	-	1.65±0.26	6.46±0.73	1.84±0.24	0.00±0.00
Cyclopropane, nonyl	-	0.00±0.00	4.96±0.56	0.00±0.00	0.00±0.00
Oxirane, (fluoromethyl)	-	0.00±0.00	2802.19±341.73	0.00±0.00	2411.48±350.73
Hexasiloxane, 1,1,3,3,5,5,7,7,9,9,11,11-dodecamethyl	-	1.59±0.24	11.16±2.13	1.48±0.26	2.65±0.34
Cyclononasiloxane, octadecamethyl	-	0.62±0.07	4.48±0.55	0.92±0.10	3.25±0.42
2,6-Dihydroxybenzoic acid, 3TMS derivative	-	1.04±0.24	2.09±0.37	0.00±0.00	2.90±0.35
1-Hexanamine, 6,N-dihydroxy	-	0.00±0.00	52.32±6.96	0.00±0.00	0.00±0.00
Thietane, 3-methyl	-	0.00±0.00	23.91±3.42	0.00±0.00	0.00±0.00
3,4-Dihydroxyphenylglycol, 4TMS derivative	-	1.27±0.24	1.77±0.28	0.79±0.08	1.69±0.27
9,12-Octadecadienoyl chloride	-	0.00±0.00	3.39±0.45	0.00±0.00	0.00±0.00
Hexadecane, 1,1-bis(dodecyloxy)	-	3.11±0.43	4.90±0.58	0.00±0.00	0.00±0.00
1,5-Dimethyl-6-oxa-bicyclo[3.1.0]hexane	-	0.00±0.00	2.60±0.38	0.00±0.00	0.00±0.00
1,4,7,10,13,16-Hexaoxacyclooctadecane	-	0.00±0.00	1.10±0.27	0.00±0.00	0.00±0.00

Table S9. Volatile flavor compounds in *Jiupei* (first fermentation)

Name	0d				10d				20d				30d			
	L1 (μg/g)	M1 (μg/g)	S1 (μg/g)	C1 (μg/g)	L1 (μg/g)	M1 (μg/g)	S1 (μg/g)	C1 (μg/g)	L1 (μg/g)	M1 (μg/g)	S1 (μg/g)	C1 (μg/g)	L1 (μg/g)	M1 (μg/g)	S1 (μg/g)	C1 (μg/g)
Ethyl Acetate	0.414	0.253	0.372	0.521	6.938	6.360	6.635	5.924	7.313	7.593	8.209	7.192	7.521	7.784	8.481	7.423
Butanoic acid ethyl ester	0.037	0.054	0.079	0.063	1.175	0.867	0.981	1.683	1.611	1.206	1.368	2.370	1.886	1.433	1.524	2.517
sec-Butyl nitrite	0.008	-	-	-	0.013	-	-	-	0.026	-	-	-	0.049	-	-	-
Methylazoxymethanol acetate	0.002	0.004	-	-	0.004	0.005	-	-	0.004	0.006	-	-	0.011	0.018	-	-
Pentanoic acid ethyl ester	0.017	0.021	0.050	0.033	0.023	0.027	0.055	0.076	0.036	0.039	0.030	0.057	0.051	0.040	0.023	0.047
Hexanoic acid ethyl ester	0.418	0.951	1.408	0.507	1.933	2.021	1.832	1.922	2.603	2.756	2.561	2.659	2.868	3.081	2.852	2.985
(S)-Isopropyl lactate	0.002	-	-	-	0.003	-	-	-	0.005	-	-	-	0.008	-	-	-
Butanoic acid, butyl ester	0.072	0.018	0.084	0.096	0.181	0.525	0.359	0.152	0.288	0.601	0.420	0.194	0.305	0.661	0.487	0.254
Heptanoic acid ethyl ester	0.009	0.003	0.005	0.008	0.014	0.010	0.017	0.018	0.031	0.017	0.034	0.044	0.065	0.028	0.052	0.056
1-Butanol, 3-methyl-, acetate	0.012	0.015	0.032	0.037	0.248	0.156	0.096	0.179	0.407	0.328	0.172	0.288	0.453	0.353	0.206	0.306
Butanoic acid, 3-methylbutyl ester	-	-	-	-	0.188	0.175	0.190	0.204	0.217	0.196	0.255	0.276	0.249	0.212	0.291	0.296
Octanoic acid ethyl ester	0.031	0.026	0.031	0.018	0.192	0.238	0.085	0.159	0.303	0.414	0.214	0.299	0.340	0.452	0.272	0.343
Butanoic acid, 2-methylpropyl ester	-	-	-	-	0.005	0.013	0.025	0.017	0.015	0.085	0.071	0.068	0.068	0.105	0.089	0.092
Nonanoic acid ethyl ester	0.008	0.009	0.014	0.002	0.011	0.029	0.047	0.019	0.084	0.084	0.097	0.071	0.105	0.120	0.123	0.133
Boronic acid, ethyl-, diethyl ester	-	-	-	-	-	-	-	-	-	0.002	-	-	-	0.008	-	-
Isoamyl lactate	-	-	-	-	-	0.005	-	0.003	-	0.007	0.007	0.029	-	0.021	0.010	0.045
Decanoic acid ethyl ester	0.012	0.024	0.028	0.018	0.112	0.098	0.142	0.179	0.153	0.128	0.205	0.217	0.178	0.145	0.185	0.201
Benzoic acid ethyl ester	0.019	0.010	0.024	0.014	0.035	0.027	0.049	0.035	0.062	0.056	0.071	0.066	0.085	0.083	0.092	0.079
Butanedioic acid diethyl ester	-	-	-	-	0.414	0.321	0.385	0.408	0.791	0.647	0.589	0.754	1.092	0.854	0.627	0.814
Acetic acid, butyl ester	-	-	-	-	-	0.026	0.034		-	0.096	0.105		-	0.119	0.128	-
Benzeneacetic acid ethyl ester	0.006	0.004	0.007	0.009	0.011	0.015	0.029	0.017	0.062	0.085	0.098	0.082	0.104	0.126	0.154	0.136
Ethyl dl-2-hydroxycaproate	-	-	-	-	-	-	-	-	-	0.009	-	-	-	0.041	-	-
Dodecanoic acid ethyl ester	-	-	-	-	-	-	-	-	0.008	-	-	-	0.019	-	-	-
Hexadecanoic acid, ethyl ester	0.079	0.079	0.018	0.099	0.217	0.252	0.204	0.136	0.395	0.443	0.357	0.330	0.428	0.462	0.403	0.381
.beta.-Phenylethyl butyrate	-	-	-	-	-	-	-	-	-	0.008	-	-	-	0.011	-	-
Methyl allylthioacetate	-	-	-	-	-	-	-	-	-	0.007	0.008	-	-	0.015	0.009	-
Tetradecanoic acid ethyl ester	-	-	-	-	0.021	0.014	-	-	0.058	0.036	-	-	0.077	0.053	-	-
9-oxo-Nonanoic acid ethyl ester	-	-	-	-	-	-	-	-	0.009	0.006	0.005	0.001	0.002	0.003	0.005	0.007

Diethyl suberate	-	-	-	-	0.118	0.107	0.080	0.104	0.197	0.152	0.107	0.156	0.172	0.171	0.122	0.186
Pentadecanoic acid ethyl ester	-	-	-	-	0.025	0.021	0.098	0.077	0.027	0.028	0.094	0.072	0.054	0.069	0.060	0.086
Diethyl azelate	-	-	-	-	0.032	0.028	0.059	0.076	0.024	0.089	0.065	0.021	0.057	0.075	0.051	0.048
2(3H)-Furanone, dihydro-5-pentyl	-	-	-	-	0.018	0.012	0.022	0.014	0.045	0.068	0.054	0.070	0.059	0.062	0.017	0.091
Hexadecanoic acid ethyl ester	0.009	0.005	0.003	0.006	0.011	0.049	0.085	0.026	0.057	0.093	0.061	0.091	0.087	0.102	0.108	0.104
Ethyl 9-hexadecenoate	-	-	-	-	-	-	-	-	0.094	0.266	0.148	0.114	0.125	0.282	0.209	0.233
Octadecanoic acid ethyl ester	-	-	-	-	0.047	0.063	-	-	0.058	0.074	0.008	0.032	0.102	0.105	0.099	0.085
(E)-9-Octadecenoic acid ethyl ester	-	-	-	-	0.039	0.060	-	-	0.170	0.108	0.008	0.006	0.203	0.142	0.030	0.052
Linoleic acid ethyl ester	-	-	-	-	0.448	0.581	0.112	0.174	0.689	0.705	0.356	0.254	0.708	0.743	0.409	0.396
(Z,Z,Z)-9,12,15-Octadecatrienoic acid, ethyl ester	-	-	-	-	0.016	-	-	-	-	0.052	-	-	0.021	0.079	-	-
9,12,15-Octadecatrienoic acid, methyl ester, (Z,Z,Z)	-	-	-	-	0.050	0.078	-	-	0.161	0.103	0.050	0.011	0.192	0.142	0.102	0.108
Acetic acid	0.009	0.007	0.004	0.006	0.428	0.540	0.573	0.521	0.699	0.707	0.798	0.723	0.863	0.871	0.827	0.927
2-methyl-Propanoic acid	0.008	-	-	-	0.010	0.018	-	-	0.096	0.099	-	-	0.114	0.126	0.094	0.080
Butanoic acid	-	-	-	-	-	0.018	-	-	-	0.085		-	0.109	0.112	0.101	0.115
Hexanoic acid	-	-	-	-	-	-	-	-	-	-	-	-	0.012	0.025	0.032	0.026
Nonanoic acid	-	-	-	-	-	-	-	-	0.045	0.061	-	0.026	0.089	0.098	0.017	0.093
Octanoic acid	-	-	-	-	-	-	-	-	-	-	-	-	0.013	0.034	0.011	0.019
2,3-dimethyl-2-Pentenoic acid	-	-	-	-	-	-	-	-	-	-	-	-	0.012	0.018	-	0.016
2-methyl-1-Propanol	0.005	0.002	0.003	0.008	0.014	0.015	0.011	0.019	0.084	0.107	0.076	0.102	0.114	0.140	0.110	0.146
1-Propanol, 2-methyl	-	-	-	-	-	0.003	-	0.004	0.008	0.021	0.007	0.012	0.014	0.042	0.012	0.054
1-Pentanol	0.194	0.185	0.122	0.183	0.392	0.426	0.425	0.322	0.590	0.602	0.618	0.495	0.621	0.649	0.655	0.583
1-Butanol, 3-methyl	0.002	-	-	-	0.009	0.007	0.005	0.008	0.011	0.012	0.014	0.021	0.091	0.034	0.070	0.081
2-Octanol	-	-	-	-	-	-	-	-	0.005	0.008	0.006	0.009	0.037	0.025	0.033	0.028
1-Heptanol	-	-	-	-	0.001	0.003	0.005	0.008	0.032	0.025	0.018	0.015	0.101	0.083	0.105	0.184
3-Octanol	-	-	-	-	-	-	-	-	0.007	0.004	0.003	0.008	0.017	0.026	0.014	0.019
2,3-Butanediol	0.004	0.008	0.005	0.009	0.025	0.040	0.012	0.038	0.061	0.144	0.052	0.072	0.106	0.161	0.109	0.175
Linalool	0.009	0.007	0.005	0.008	0.014	0.020	0.019	0.025	0.058	0.062	0.071	0.057	0.093	0.098	0.084	0.087
1-Hexanol, 2-ethyl	-	-	-	-	-	-	-	-	-	-	-	-	0.004	0.007	0.005	0.006
1-Nonanol	-	-	-	-	-	-	-	-	0.003	0.006	0.002	0.005	0.017	0.028	0.012	0.011
1-Decanol	-	-	-	-	-	-	-	-	-	-	-	-	0.002	0.004	0.002	0.004
3-(methylthio)-1-Propanol	-	-	-	-	-	-	-	-	-	-	-	-	0.005	0.008	0.004	0.005
Benzyl alcohol	-	-	-	-	0.005	0.006	0.001	0.005	0.019	0.041	0.013	0.027	0.095	0.106	0.090	0.101
Phenylethyl alcohol	0.004	0.005	0.004	0.007	0.018	0.021	0.016	0.020	0.067	0.071	0.063	0.069	0.112	0.126	0.169	0.170

5-Decen-1-ol, (E)-	-	-	-	-	-	-	-	-	0.006	0.005	0.007	0.004	0.019	0.023	0.028	0.022
Hexanal	-	-	-	-	-	-	-	-	0.003	0.005	0.008	0.001	0.031	0.021	0.028	0.011
Decanal	0.002	0.004	0.005	0.007	0.018	0.091	0.016	0.022	0.071	0.072	0.085	0.062	0.140	0.119	0.163	0.140
2-Heptenal	0.005	0.007	0.002	0.006	0.015	0.019	0.027	0.011	0.066	0.074	0.069	0.081	0.129	0.114	0.137	0.101
2-Undecenal	0.006	0.001	0.004	0.008	0.013	0.020	0.017	0.029	0.066	0.078	0.043	0.069	0.113	0.139	0.139	0.129
Furfural	-	-	-	-	-	-	-	-	-	-	-	-	0.006	0.009	0.001	0.002
Benzaldehyde	0.006	0.007	0.005	0.002	0.011	0.023	0.051	0.016	0.055	0.070	0.089	0.074	0.103	0.123	0.160	0.119
Pentanal	-	-	-	-	-	-	-	-	0.001	0.008	0.007	0.003	0.013	0.019	0.021	0.014
Benzeneacetaldehyde	0.007	0.006	0.001	0.003	0.021	0.032	0.029	0.014	0.054	0.069	0.049	0.058	0.094	0.077	0.082	0.089
2-Octanone	0.002	0.009	0.001	0.005	0.016	0.010	0.025	0.021	0.044	0.033	0.058	0.039	0.084	0.045	0.094	0.082
Acetoin	-	-	-	-	-	-	-	-	-	-	-	-	0.006	0.002	0.008	0.004
Undecane	0.008	0.001	0.004	0.002	0.017	0.023	0.014	0.036	0.056	0.058	0.063	0.068	0.085	0.084	0.092	0.099
Styrene	-	-	-	-	-	-	-	-	0.003	0.005	0.003	0.007	0.041	0.030	0.046	0.051
Tridecane	-	-	-	-	-	-	-	-	0.007	0.004	0.005	0.006	0.035	0.024	0.019	0.014
Tetradecane	-	-	-	-	0.002	0.007	0.009	0.003	0.027	0.024	0.030	0.029	0.045	0.054	0.049	0.038
Cyclopentasiloxane, decamethyl	0.009	0.003	0.009	0.006	0.022	0.012	0.035	0.017	0.050	0.044	0.062	0.045	0.079	0.089	0.084	0.096
Hexadecane					0.005	0.004	0.003	0.007	0.024	0.033	0.017	0.015	0.065	0.073	0.064	0.074
1-Pentadecene	0.005	0.002	0.004	0.008	0.028	0.019	0.015	0.021	0.054	0.048	0.059	0.049	0.081	0.073	0.083	0.096
Cyclohexasiloxane, dodecamethyl	-	-	-	-	-	-	-	-	-	-	-	-	0.005	0.008	0.007	0.004
Phenol	-	-	-	-	0.004	0.002	0.001	0.007	0.018	0.024	0.016	0.025	0.071	0.084	0.075	0.078
4-ethyl-2-methoxy-Phenol	-	-	-	-	-	-	-	-	0.007	0.006	0.001	0.004	0.016	0.036	0.023	0.041
4-ethyl-Phenol	-	-	-	-	0.006	0.004	0.008	0.005	0.018	0.021	0.020	0.015	0.047	0.036	0.037	0.049
2,3-dihydro-Benzofuran	-	-	-	-	-	-	-	-	0.007	0.003	0.007	0.002	0.017	0.014	0.019	0.028
2-(Allylthio)acetonitrile	-	-	-	-	-	-	-	-	0.003	0.002	0.005	0.008	0.014	0.024	0.023	0.015
Pyridine, 2,6-diamino-3-((2,5-dichloropenyl)azo)	-	-	-	-	0.007	0.001	0.005	0.009	0.017	0.021	0.027	0.018	0.039	0.043	0.046	0.008

Table S10. Volatile flavor compounds in *Jiupei* (second fermentation)

Name	0d				10d				20d				30d			
	L2 (μg/g)	M2 (μg/g)	S2 (μg/g)	C2 (μg/g)	L2 (μg/g)	M2 (μg/g)	S2 (μg/g)	C2 (μg/g)	L2 (μg/g)	M2 (μg/g)	S2 (μg/g)	C2 (μg/g)	L2 (μg/g)	M2 (μg/g)	S2 (μg/g)	C2 (μg/g)
Ethyl Acetate	3.251	2.008	3.273	2.741	8.182	8.319	7.907	7.090	10.951	11.996	9.679	9.118	12.542	13.665	10.408	9.649
Butanoic acid ethyl ester	0.628	0.304	0.685	0.471	2.573	2.819	2.086	1.724	2.988	3.789	2.841	2.205	3.846	4.416	3.143	2.952
sec-Butyl nitrite	0.021	0.030	0.015	0.033	0.079	0.119	0.083	0.098	0.119	0.187	0.115	0.124	0.186	0.241	0.148	0.140
Methylazoxymethanol acetate	-	-	-	-	0.001	0.003	0.005	0.002	0.042	0.072	0.057	0.049	0.086	0.098	0.081	0.068
Pentanoic acid ethyl ester	0.032	0.044	0.025	0.038	0.080	0.107	0.074	0.089	0.141	0.169	0.113	0.126	0.182	0.194	0.138	0.135
Hexanoic acid ethyl ester	1.481	1.035	1.976	1.394	3.207	3.628	2.230	2.017	3.977	5.273	3.618	3.050	4.924	5.552	3.897	3.199
(S)-Isopropyl lactate	-	-	-	-	-	-	-	-	0.005	0.008	0.006	0.004	0.019	0.025	0.018	0.024
Butanoic acid, butyl ester	0.176	0.122	0.153	0.171	0.708	0.772	0.705	0.659	0.990	1.049	0.956	0.904	1.165	1.387	1.197	1.046
Heptanoic acid ethyl ester	0.016	0.013	0.022	0.035	0.094	0.147	0.087	0.104	0.131	0.185	0.134	0.127	0.192	0.256	0.169	0.153
1-Butanol, 3-methyl-, acetate	0.102	0.106	0.117	0.125	1.112	1.043	0.965	0.906	1.598	1.775	1.451	1.403	1.878	1.977	1.610	1.529
Butanoic acid, 3-methylbutyl ester	0.148	0.121	0.133	0.170	0.623	0.599	0.604	0.565	0.984	0.942	0.877	0.811	1.105	1.179	1.054	1.006
Octanoic acid ethyl ester	0.114	0.115	0.120	0.149	0.791	0.929	0.801	0.786	1.072	1.389	1.162	1.115	1.426	1.606	1.245	1.210
Butanoic acid, 2-methylpropyl ester	0.022	0.043	0.050	0.041	0.115	0.316	0.147	0.145	0.154	0.697	0.200	0.382	0.275	0.833	0.258	0.393
Nonanoic acid ethyl ester	0.026	0.037	0.049	0.035	0.438	0.429	0.491	0.403	0.812	0.727	0.784	0.680	0.912	0.943	0.905	0.707
Boronic acid, ethyl-, diethyl ester	-	-	-	-	0.024	0.016	0.037	-	0.050	0.059	0.068	-	0.076	0.085	0.080	-
Isoamyl lactate	0.012	0.023	0.028	0.016	0.074	0.099	0.076	0.095	0.097	0.128	0.095	0.108	0.144	0.159	0.119	0.113
Decanoic acid ethyl ester	0.142	0.120	0.169	0.157	0.701	0.806	0.713	0.697	0.989	0.993	0.904	1.085	1.237	1.276	1.146	1.108
Benzoic acid ethyl ester	0.041	0.043	0.050	0.055	0.135	0.234	0.149	0.148	0.394	0.445	0.392	0.327	0.485	0.549	0.473	0.420
Butanedioic acid diethyl ester	0.291	0.112	0.291	0.228	1.454	1.558	1.109	1.059	2.518	2.720	1.913	1.908	2.879	3.008	2.218	2.069
Acetic acid, butyl ester	0.043	0.075	0.061	0.038	0.380	0.509	0.409	0.396	0.675	0.867	0.708	0.715	0.975	1.027	0.942	0.810
Benzeneacetic acid ethyl ester	0.033	0.042	0.058	0.028	0.408	0.645	0.550	0.413	0.799	0.862	0.786	0.706	0.976	1.103	1.001	0.819
Dodecanoic acid, ethyl ester	-	-	-	-	0.013	0.022	0.019	0.024	0.073	0.096	0.065	0.080	0.099	0.126	0.084	0.103
Dodecanoic acid ethyl ester	-	-	-	-	0.015	0.013	0.020	0.018	0.085	0.071	0.064	0.066	0.102	0.099	0.081	0.086
Hexadecanoic acid, ethyl ester	0.135	0.159	0.216	0.230	0.946	0.907	0.814	0.952	1.371	1.285	1.199	1.059	1.578	1.506	1.349	1.211
.beta.-Phenylethyl butyrate	-	-	-	-	0.005	0.002	0.008	0.006	0.035	0.029	0.037	0.021	0.064	0.056	0.079	0.060
Methyl allylthioacetate	-	-	-	-	-	-	-	-	0.020	0.028	0.026	0.024	0.051	0.069	0.045	0.057
Tetradecanoic acid ethyl ester	0.034	0.027	0.026	0.032	0.078	0.085	0.067	0.070	0.104	0.109	0.099	0.112	0.120	0.154	0.115	0.117
9-oxo-Nonanoic acid ethyl ester	0.001	0.003	0.003	0.004	0.007	0.004	0.005	0.005	0.011	0.014	0.010	0.009	0.008	0.006	0.009	0.008

Diethyl suberate	0.087	0.091	0.082	0.095	0.159	0.189	0.140	0.124	0.214	0.225	0.168	0.179	0.251	0.277	0.199	0.208
Pentadecanoic acid ethyl ester	0.022	0.025	0.037	0.040	0.041	0.048	0.053	0.068	0.066	0.075	0.067	0.081	0.087	0.099	0.079	0.091
Diethyl azelate	0.041	0.032	0.047	0.037	0.055	0.059	0.062	0.060	0.068	0.076	0.081	0.074	0.079	0.088	0.094	0.080
2(3H)-Furanone, dihydro-5-pentyl	0.019	0.020	0.016	0.022	0.041	0.052	0.046	0.059	0.055	0.068	0.061	0.072	0.067	0.075	0.070	0.080
Hexadecanoic acid ethyl ester	0.011	0.020	0.041	0.036	0.031	0.069	0.085	0.062	0.052	0.080	0.092	0.091	0.068	0.088	0.090	0.094
Ethyl 9-hexadecenoate	-	-	-	-	0.010	0.014	0.025	0.018	0.038	0.055	0.042	0.045	0.050	0.064	0.061	0.058
Octadecanoic acid ethyl ester	0.032	0.041	0.029	0.036	0.062	0.069	0.059	0.066	0.089	0.090	0.081	0.076	0.113	0.124	0.105	0.099
(E)-9-Octadecenoic acid ethyl ester	0.038	0.045	0.050	0.048	0.108	0.100	0.097	0.120	0.160	0.158	0.143	0.145	0.175	0.184	0.160	0.168
Linoleic acid ethyl ester	0.724	0.736	0.641	0.769	1.139	1.213	1.067	1.025	1.373	1.566	1.396	1.372	1.621	1.718	1.513	1.501
Tetradecanoic acid, ethyl ester	-	-	-	-	0.018	0.020	0.021	-	0.033	0.047	0.036	-	0.039	0.054	0.047	-
9,12,15-Octadecatrienoic acid, methyl ester, (Z,Z,Z)	0.052	0.043	0.057	0.049	0.114	0.107	0.123	0.108	0.161	0.153	0.150	0.171	0.181	0.175	0.185	0.197
Acetic acid	0.072	0.080	0.091	0.065	0.986	1.254	0.881	1.012	1.769	1.897	1.488	1.406	1.975	2.285	1.749	1.713
2-methyl-Propanoic acid	0.088	0.075	0.069	0.062	0.110	0.148	0.117	0.108	0.166	0.199	0.170	0.164	0.189	0.226	0.194	0.180
Butanoic acid	0.070	0.081	0.062	0.059	0.127	0.138	0.116	0.114	0.170	0.195	0.157	0.169	0.192	0.202	0.181	0.195
Hexanoic acid	0.012	0.010	0.017	0.012	0.052	0.049	0.056	0.055	0.070	0.081	0.072	0.069	0.085	0.097	0.083	0.080
Nonanoic acid	0.022	0.026	0.035	0.028	0.065	0.055	0.048	0.051	0.080	0.088	0.069	0.073	0.092	0.108	0.087	0.093
Octanoic acid	0.009	0.010	0.008	0.011	0.035	0.050	0.056	0.060	0.044	0.071	0.072	0.077	0.064	0.084	0.081	0.089
8-Methylnonanoic acid	-	-	-	-	-	-	-	-	0.008	0.009	0.010	0.012	0.022	0.028	0.027	0.026
2-methyl-1-Propanol	0.035	0.032	0.023	0.028	0.114	0.145	0.111	0.109	0.164	0.197	0.166	0.172	0.199	0.220	0.180	0.196
1-Propanol, 2-methyl	0.009	0.013	0.008	0.014	0.045	0.043	0.039	0.054	0.069	0.071	0.057	0.072	0.084	0.082	0.072	0.084
1-Pentanol	0.191	0.172	0.292	0.115	0.874	1.080	0.763	0.704	1.585	1.787	1.349	1.306	1.956	2.141	1.553	1.582
1-Butanol, 3-methyl	0.015	0.016	0.014	0.019	0.089	0.097	0.085	0.098	0.121	0.112	0.104	0.141	0.151	0.134	0.120	0.161
2-Octanol	0.006	0.009	0.008	0.010	0.044	0.037	0.035	0.038	0.065	0.058	0.056	0.049	0.072	0.065	0.063	0.068
1-Heptanol	0.030	0.025	0.021	0.018	0.101	0.113	0.095	0.108	0.142	0.165	0.148	0.145	0.171	0.183	0.165	0.164
3-Octanol	-	-	-	-	0.012	0.018	0.010	0.012	0.027	0.026	0.023	0.028	0.037	0.036	0.034	0.039
2,3-Butanediol	0.044	0.048	0.045	0.049	0.095	0.110	0.072	0.108	0.131	0.154	0.112	0.142	0.166	0.181	0.139	0.165
Linalool	0.039	0.037	0.045	0.038	0.084	0.102	0.099	0.075	0.118	0.132	0.121	0.097	0.134	0.158	0.144	0.127
1-Hexanol, 2-ethyl	-	-	-	-	0.002	0.005	0.005	0.004	0.022	0.020	0.032	0.027	0.035	0.037	0.041	0.036
1-Nonanol	0.007	0.09	0.006	0.005	0.038	0.045	0.036	0.044	0.067	0.076	0.059	0.071	0.088	0.098	0.072	0.091
1-Decanol	-	-	-	-	-	-	-	-	0.005	0.008	0.006	0.007	0.022	0.024	0.019	0.024
3-(methylthio)-1-Propanol	-	-	-	-	-	-	-	-	0.002	0.007	0.004	0.009	0.021	0.028	0.024	0.025
Benzyl alcohol	0.015	0.020	0.017	0.019	0.064	0.086	0.061	0.075	0.099	0.108	0.086	0.097	0.112	0.136	0.104	0.121
Phenylethyl alcohol	0.024	0.025	0.027	0.037	0.118	0.127	0.116	0.120	0.157	0.171	0.163	0.149	0.187	0.196	0.190	0.170

5-Decen-1-ol, (E)-	-	-	-	-	0.004	0.005	0.008	0.005	0.036	0.055	0.047	0.044	0.054	0.073	0.068	0.062
Hexanal	0.012	0.010	0.016	0.006	0.034	0.020	0.029	0.036	0.048	0.040	0.053	0.059	0.061	0.051	0.068	0.071
Decanal	0.020	0.024	0.023	0.027	0.108	0.111	0.116	0.102	0.164	0.156	0.162	0.151	0.182	0.179	0.183	0.170
2-Heptenal	0.024	0.027	0.020	0.025	0.105	0.119	0.107	0.101	0.159	0.152	0.145	0.163	0.172	0.179	0.167	0.181
2(3H)-Furanone, dihydro-5-pentyl-	0.025	0.021	0.024	0.029	0.103	0.120	0.117	0.118	0.156	0.168	0.143	0.169	0.190	0.199	0.177	0.189
Furfural	-	-	-	-	0.002	0.004	0.005	0.004	0.015	0.010	0.023	0.027	0.026	0.019	0.031	0.042
Benzaldehyde	0.036	0.027	0.035	0.042	0.111	0.123	0.104	0.117	0.164	0.151	0.138	0.166	0.183	0.173	0.170	0.199
Pentanal	0.010	0.014	0.012	0.012	0.033	0.038	0.029	0.026	0.041	0.052	0.047	0.048	0.053	0.069	0.061	0.064
Benzeneacetaldehyde	0.027	0.025	0.032	0.033	0.072	0.068	0.063	0.079	0.095	0.089	0.101	0.108	0.112	0.107	0.122	0.139
2-Octanone	0.021	0.025	0.031	0.027	0.061	0.072	0.065	0.068	0.088	0.093	0.085	0.096	0.104	0.115	0.110	0.112
Acetoin	-	-	-	-	0.005	0.007	0.005	0.004	0.014	0.023	0.016	0.013	0.026	0.032	0.028	0.024
Undecane	0.024	0.031	0.027	0.032	0.071	0.076	0.060	0.067	0.096	0.108	0.083	0.098	0.115	0.124	0.102	0.119
Styrene	0.013	0.014	0.022	0.020	0.045	0.053	0.040	0.049	0.068	0.073	0.063	0.065	0.082	0.092	0.076	0.081
Tridecane	0.012	0.010	0.008	0.007	0.041	0.049	0.053	0.055	0.062	0.069	0.066	0.070	0.077	0.084	0.089	0.085
Tetratetracontane	0.022	0.028	0.030	0.025	0.050	0.048	0.045	0.041	0.067	0.066	0.064	0.068	0.082	0.079	0.080	0.075
Cyclopentasiloxane, decamethyl	0.011	0.015	0.019	0.016	0.055	0.064	0.058	0.063	0.079	0.088	0.074	0.089	0.093	0.105	0.093	0.109
Hexadecane	0.020	0.021	0.031	0.027	0.058	0.064	0.069	0.077	0.065	0.057	0.065	0.069	0.088	0.093	0.084	0.083
1-Pentadecene	0.016	0.022	0.018	0.028	0.075	0.069	0.077	0.081	0.094	0.098	0.089	0.095	0.102	0.114	0.108	0.106
Cyclohexasiloxane, dodecamethyl		-	-	-	-	-	-	-	0.004	0.005	0.002	0.003	0.015	0.016	0.017	0.014
Phenol	0.016	0.017	0.023	0.020	0.066	0.062	0.061	0.062	0.089	0.094	0.086	0.095	0.105	0.104	0.107	0.108
4-ethyl-2-methoxy-Phenol	-	-	-	-	-	-	-	-	0.012	0.014	0.011	0.013	0.026	0.029	0.031	0.045
4-ethyl-Phenol	0.021	0.025	0.026	0.020	0.055	0.059	0.048	0.065	0.070	0.071	0.072	0.075	0.089	0.096	0.087	0.088
Phenol, 4-ethyl-2-methoxy-	-	-	-	-	-	-	-	-	0.012	0.013	0.015	0.012	0.020	0.024	0.026	0.031
2-(Allylthio)acetonitrile	-	-	-	-	-	-	-	-	0.013	0.015	0.017	0.018	0.024	0.030	0.028	0.025
Pyridine, 2,6-diamino-3-((2,5-dichloropenyl)azo)	-	-	-	-	-	-	-	-	0.022	0.028	0.033	0.028	0.042	0.041	0.048	0.038
